# HSP70 Family in Cancer: Signaling Mechanisms and Therapeutic Advances

**DOI:** 10.3390/biom13040601

**Published:** 2023-03-27

**Authors:** Kejia Zhao, Guanyu Zhou, Yu Liu, Jian Zhang, Yaohui Chen, Lunxu Liu, Gao Zhang

**Affiliations:** 1 Department of Thoracic Surgery and Institute of Thoracic Oncology, West China Hospital, Sichuan University, Chengdu 610041, China; 2 Western China Collaborative Innovation Center for Early Diagnosis and Multidisciplinary Therapy of Lung Cancer, Chengdu 610041, China; 3 Frontiers Science Center for Disease-related Molecular Network, West China Hospital, Sichuan University, Chengdu 610041, China; 4 Faculty of Dentistry, The University of Hong Kong, Prince Philip Dental Hospital, Hong Kong 999077, China

**Keywords:** HSP70, cancer, signaling pathway, treatment

## Abstract

The 70 kDa heat shock proteins (HSP70s) are a group of highly conserved and inducible heat shock proteins. One of the main functions of HSP70s is to act as molecular chaperones that are involved in a large variety of cellular protein folding and remodeling processes. HSP70s are found to be over-expressed and may serve as prognostic markers in many types of cancers. HSP70s are also involved in most of the molecular processes of cancer hallmarks as well as the growth and survival of cancer cells. In fact, many effects of HSP70s on cancer cells are not only related to their chaperone activities but rather to their roles in regulating cancer cell signaling. Therefore, a number of drugs directly or indirectly targeting HSP70s, and their co-chaperones have been developed aiming to treat cancer. In this review, we summarized HSP70-related cancer signaling pathways and corresponding key proteins regulated by the family of HSP70s. In addition, we also summarized various treatment approaches and progress of anti-tumor therapy based on targeting HSP70 family proteins.

## 1. Introduction

The history of the first discovery of heat shock proteins (HSPs) can be dated to 1962 in Ferruccio Ritossa’s lab. They found a “puffing pattern”, which indicated elevated gene transcription of some unknown proteins after a colleague accidentally raised the incubation temperature of Drosophila [1]. In the next two decades, several studies further demonstrated that the main function of these “unknown proteins” is to protect cells from various non-lethal heat shock or other stimuli [2,3,4,5,6]. Based on these results, this phenomenon was described as the “Heat Shock Response”, and the translated unknown proteins were termed “heat shock proteins” [7]. A group of 70 kDa proteins were initially classified as the HSP70 family according to their molecular weight. However, with the development of sequencing technology, some genes with similar sequence structure were also included in the HSP70 family, which brings to a total of 13 homologues of the HSP70 family in homo sapiens currently [8]. The main function of HSP70 consists of two parts. First, the house-keeping activities contain de novo protein folding, protein translocation across membranes, disassembly of protein complexes, and regulation of protein activities. Second, the stress-related activities maintain protein stability under stressful situations, including the prevention of protein aggregation, disaggregation, refolding, and degradation [9]. Molecular mechanisms and working principles of the HSP70 network were reviewed in detail in Ref [9,10]. Notably, dysregulation of the HSP70 network results in important consequences in multiple aspects of cancer development and progression [11,12]. Multiple studies have already revealed that expression of HSP70 at a higher level was detected in many cancer types and associated with poor prognosis, recurrence, and treatment resistance [13,14,15,16,17]. Therefore, research on the role of HSP70 in tumors, such as its binding site, substrate proteins, and related pathways, is in continuous progress, and relevant research on potential therapies is also underway. In this review, we first summarized common cancer related signaling pathways regulated by the HSP70 family proteins such as the RTKs-RAS-RAF-MEK-ERK pathway, the PI3K/AKT/mTOR pathway, and key proteins of other signaling pathways, in a direct or indirect manner. In addition, we also summarized HSP70-based monotherapy, combination therapy, HSP70 as an adjuvant in cancer vaccine therapies, and related clinical trials.

## 2. HSP70 Family: Family Members, Common Structure, and Basic Function

The human HSP70 family has 13 homologues [18]. We searched their official name, aliases, cell location, and other basic information from HUGO Gene Nomenclature Committee (HGNC), National Center for Biotechnology Information (NCBI), and The Human Protein Atlas database. Related information was summarized in Supplementary Table S1. 

**Figure 1:**
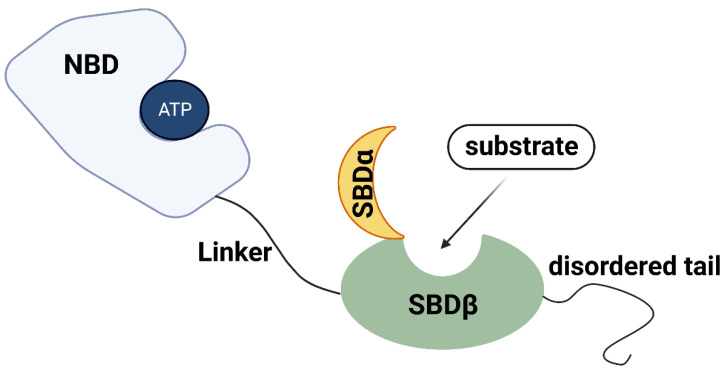
Schematic diagram of HSP70 structure domain. A typical HSP70 domain structure consists of NBD, linker, SBD, and a disordered tail. NBD binds and hydrolyzes ATP to control the lobe movements. Linker is essential for the NBD conformational changing. SBD has two functional parts and works together on substrate binding. The disordered tail interacts with specific cofactors to fulfil HSP70 functions. Nucleotide-binding domain (NBD). Substrate-binding domain (SBD).

The HSP70 family is a group of highly conserved molecules in both prokaryotes and eukaryotes [19,20]. A typical HSP70 domain structure consists of the following components and annotates in Figure 1. The nucleotide-binding domain (NBD), located in the N-terminal of HSP70, is composed of four subdomains (IA, IB, IIA, IIB) and is arranged into two lobes separated by a deep cleft in the middle [9,18]. The main function of the NBD domain is to bind and hydrolyze ATP to control the lobe movements [21,22]. A highly conserved hydrophobic linker, which is essential for the NBD conformational changing when ATP binds to NBD, connects NBD with the C-terminal substrate-binding domain (SBD) [19,20,21,23]. The SBD can be further divided into two functional parts, a N-terminal β-sheet subdomain (SBDβ) and a C-terminal α-helical subdomain (SBDα). SBDβ is built up by an eight-stranded β-sandwich constituted of a substrate binding groove, and SBDα is built up by α-helixes which act as a flexible lid [24]. The function of SBD is closely related to the state of ATP/ADP binding to NBD. When ATP binds to NBD, the interdomain linker and SBDα/β work together on NBD conformational changing, making the NBD unsuitable for ATP hydrolysis [25]. After substate binding to the hydrophobic pocket of SBDβ, the SBDα/β are released from NBD, resulting in the recovery of NBD ATPase activity [25,26]. The SBDα lid is then closed to prevent substrate dissociation [24,27]. When NBD switches to the ADP binding state, the SBD binds to substrates with a high affinity and slow association and dissociation rates [9]. The release of substrates is also based on the conformational changes of HSP70 when recombined with ATP [9]. Furthermore, a disordered tail, located behind the SBD, contains an EEVD (Glu-Glu-Val-Asp) motif that interacts with specific cofactors to fulfil HSP70 functions [28,29,30]. The HSP70 chaperones are required to work with other co-chaperones to carry out its full function cycle [9,10]. The two most important co-chaperones are HSP40, also known as J-domain proteins (JDPs), and nucleotide exchange factors (NEFs) [31,32]. In general, JDPs deliver substrates to HSP70 and stimulate the ATPase domain, whereas NEFs induce substrate release and rebinding of ATP.

## 3. HSP70 Regulates Multiple Cancer Related Signaling Pathways

**Figure 2:**
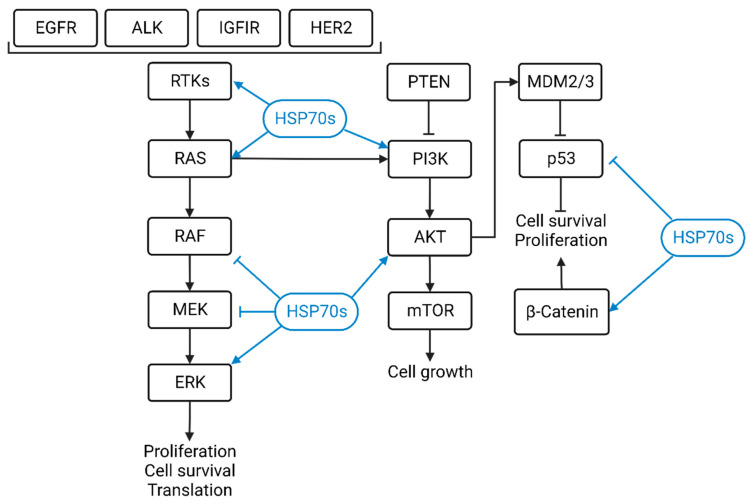
Schematic diagram of cancer related signaling pathways regulated by HSP70s. HSP70 family members regulate important phenotypes such as tumor cell survival and proliferation by regulating a variety of key proteins in cancer-related signaling pathways.

HSP70 participates in wide range of cancer development and progression through dysregulating multiple cancer-related signaling pathways. In this review, we summarized the function of HSP70 in frequently altered oncogenic signaling pathways in cancer (Figure 2).

### 3.1. RTKs-RAS-RAF-MEK-ERK Pathway

Receptor tyrosine kinases (RTKs) are important receptor proteins on the tumor cell membrane that initiate activation of cancer signaling pathways. Much evidence implicates the role of HSP70 in regulating a variety of RTKs. Epidermal growth factor receptor (EGFR) could be activated by extracellular HSPBP1 and HSPA1A/B in a synergistically way [33]. Secreted HSPA5 also activated EGFR signaling and conferred the resistance of hepatocellular carcinoma (HCC) cells to sorafeinib [34]. Mechanically, it promoted phosphorylation and activation of insulin-like growth factor I receptor (IGF-IR) to facilitate cell proliferation and migration [35]. HSPA1A/B assisted folding of oncogenic nucleophosmin–anaplastic lymphoma kinase (NPM-ALK) in anaplastic large-cell lymphomas (ALCLs) and maintained its malignant phenotype [36,37]. Moreover, it played an essential role in Her2-induced mammary tumorigenesis in which HSP72-depleted cells caused cellular senescence and failed to induce neoplastic transformation [38]. Additionally, HSP70 can exert its influence on the RAS pathway by regulating KRAS. HSPA5 haploinsufficiency suppressed both KRAS(G12D)-driven pancreatic and lung tumorigenesis [39]. Furthermore, knockdown of HSPA5 via siRNA reduced the oncogenic KRAS protein level in various KRAS mutated cancers [40,41,42]. HSP70 inhibited the downstream signaling molecules of RAF [43]. Bag1 was bound to and activated Raf-1, subsequently activating the downstream extracellular signal-related kinases (ERKs) [43]. However, HSP70 may compete for binding to Bag1, which indirectly leads to the inhibition of RAF activation [43]. The downstream signaling molecules of MEK were inhibited by HSPA9, which facilitated protein phosphatase 1α (PP1α)-mediated MEK1/2 dephosphorylation by promoting the interaction of MEK1/2 with PP1α in an ATP-sensitive manner [44]. Though lots of evidence showed that the HSP70 family, especially HSPA5, indirectly promoted the expression of ERK, whether HSP70 has a direct effect on ERK still remains unclear and needs to be further explored [45,46,47,48,49,50,51,52]. Nevertheless, the majority of studies suggest that HSP70 plays an activating role in regulating the RTKs-RAS-RAF-MEK-ERK signaling pathway.

### 3.2. PI3K/AKT/mTOR Pathway

The PI3K/AKT/mTOR pathway is activated in a wide type of cancers, leading to tumor proliferation and therapeutic resistance [53]. HSP70 induces PI3K activation in many cancer types [45,54,55,56,57]. Overexpression of HSPA5 promoted PIP3 formation and PI3K activation through forming a complex with PI3K [58]. Using an inhibitor or monoclonal antibody of HSPA5 can inhibit the PI3K pathway and suppress tumor growth and metastasis [57,59]. Besides, knock-out HSPA5 in PTEN-null background suppressed the activation of PI3K downstream protein AKT in a variety of disease models [60,61,62]. HSPA9 also facilitated PI3K/AKT signaling, thus promoting cancer progression [61,63].

HSP70 generally tends to promote AKT [57,61,63]; however, in some circumstances it may interfere with AKT. The arginylated form of HSPA5 binds with fully ubiquitated AKT (K284 to K214) and induces AKT degradation via the autophagy-lysosome pathway [64]. The PI3K signaling pathway regulated by HSP70 can be transmitted to mTOR in most circumstances [59,65]. However, Ryu et al. showed that HSP70 comes into contact with Rheb and inhibits the mTORC1 signaling pathway [66]. However, the evidence of how HSP70 inhibits the mTORC1 signaling pathway is lacking. Therefore, we concluded that HSP70 tends to promote the PI3K/AKT/mTOR signaling pathway in general.

### 3.3. Effect of HSP70 on Key Proteins of Other Signaling Pathways 

**P53**

The effect of HSP70 on p53 function was initially controversial. Some studies claimed that HSP70 was preferred to maintain the stability of p53-wild type (WT) at higher temperatures and support its DNA-binding [67,68,69]. Furthermore, the mutant p53 protein half-life can be increased by HSP70- and MDM2-dependent protein co-aggregates [70]. On the contrary, others found that HSP70 could sequester p53 in the cytoplasm and negatively regulated its stability [71,72]. These contradictory results make the role of HSP70 confusing with regard to regulating the function of p53 protein. Until recently, two independent teams both demonstrated that HSP70 inactivated p53 at physiological temperatures by unfolding p53. Boysen and colleagues found that HSP70, together with J-domain protein Hdj1 and ATP, increases local unfolding in both WT and mutant p53-DNA binding domain and dissociates p53 from DNA by binding to its DNA binding loop [73]. This was also proved by Dahiya and colleagues that HSP70 inactivates WT-p53 at physiological temperatures by unfolding it. The above two independent team unanimously pointed out that it was HSP90 not HSP70 that promotes the folding of the p53 protein in an ATP-dependent manner [74]. Thus, the HSP70 and HSP90 chaperone systems assume complementary functions to optimally balance conformational plasticity with p53 conformational stability [73,74]. Based on these results, HSP70 itself tends to negatively regulate both WT and the mutant p53 protein. 

**β-catenin**

Lots of evidence suggested that HSP70 was involved in regulating the β-catenin signaling pathway. HSPA5 was initially found to play an important role in regulating the β-catenin signaling axis through interacting with β-catenin [75]. Li and colleagues further found that HSPA5 enhanced β-catenin signaling and consequently promoted its downstream c-Myc-mediated glutamine metabolism in colorectal cancer cells [76]. Meanwhile, HSPA9 could maintain the stemness of breast cancer stem cells via activating the Wnt/GSK3β/β-catenin signaling pathway [77]. Furthermore, overexpression of HSPA1A could also facilitate the activation of the Wnt/β-catenin signaling pathway, resulting in the enhancement of osteogenic differentiation of bone marrow mesenchymal stem cells [78]. 

**SMAD and NF-κB**

The role of HSP70 in regulating SMAD proteins and the nuclear factor kappa B (NF-κB) function remains controversial. HSPA8 was reported to activate TGF-β-induced Smad signaling through functional interaction with Smad2/3 [79]. Overexpression of HSPA5 up-regulated the expression and secretion of TGF-β1, which further promotes the cell-matrix adhesion and epithelial-mesenchymal transition (EMT) through activating the downstream Smad2/3 signaling module [80]. Moreover, a blockade of Cripto interacting with the cell surface HSPA5 suppressed oncogenic Cripto signaling via Smad2/3 pathways [45]. However, other researchers also showed that HSP70 exerted an anti-activity function in SMAD proteins. HSP70 decreased receptor-dependent phosphorylation of Smad2 and blocked TGF-β-induced EMT [81]. The inhibitory effect of HSP70 was also exhibited by the activation of Smad3 induced by high level of glucose [82]. HSPA1A/B inhibited Smad3 activation and nuclear translocation in renal EMT [83]. In terms of the NF-κB pathway, membrane-bound HSP70 was found to induce transcription NF-κB whereas HSP70 in the cytoplasm may repress NF-κB expression [84]. This was also proved by Sheppard et al., who found that HSP70 in the cytoplasm blocked NF-κB activation by inhibiting IKK [85].

## 4. Targeting HSP70 in Cancer Therapy

Given that HSP70 exerts an important function in multiple aspects of cancer development and progression, major endeavors have been focused on the development of therapies targeting HSP70 in cancer over the last 30 years. Roughly, they can be divided into two directions: one is to identify inhibitors targeting HSP70 based on its cancer-promoting role and the other is to develop cancer vaccines in which HSP70 serves as an adjuvant based on its immunostimulatory effect. Though no HSP70 inhibitors or -based vaccines have been approved by the FDA to date, corresponding preclinical studies and clinical trials are still ongoing, and many new research directions, such as exploring the combination therapeutic mode of HSP70 inhibitors with chemotherapy, radiotherapy, or targeted therapy, are also being attempted.

### 4.1. HSP70 as an Inhibitory Target in Cancer Therapies

#### 4.1.1. HSP70 Inhibitors in Monotherapy Mode

We catalogued the currently published HSP70 inhibitors according to their binding sites on HSP70 and summarized their chemical structures, effects on HSP70 activity, and applications in cancer therapeutics, including cancer types, effects on cancers, and corresponding mechanisms, in Table 1.

##### NBD-Binding Inhibitors

NBD-binding inhibitors were identified or developed to interfere with the function of HSP70 through inhibiting ATPase activity of HSP70 or affecting the binding of nucleotide exchange factors (NEFs) or J domain protein (JDP) to HSP70. VER-155008, the most typical example of ATP-competitive HSP70 inhibitors, is a derivative of ATP. Through interacting with the ATP binding site, HSP70 inhibits ATPase activity of all isoforms of HSP70 [127,128,132,133,134]. Though this lacking isoform specificity, VER-155008 was identified to exert strong anti-tumor activity in a variety of cancers through affecting a wide range of cancer-related signaling pathways [59,88,89,90,91,92,93,94]. VER-155008 inhibited proliferation, suppressed invasion and migration, and induced apoptosis of pheochromocytoma cells through down-regulating phosphorylation of the PI3K/AKT/mTOR and MEK/ERK signaling pathways [59]. In castration-resistant prostate cancer (CRPC) cells, VER-155008 suppressed the expression of full-length androgen receptor (AR) and AR splice variant 7 (ARv7) through Y-box binding protein 1 (YB-1) inhibition, which makes it an attractive anti-tumor agent for treating CRPC [91,92]. Another well-studied ATP-competitive HSP70 inhibitor Apoptozole displayed toxicity against a broad spectrum of cancer cells, such as oral squamous cell carcinoma, breast cancer, and liver cancer cells. Furthermore, Apoptozole suppressed tumor growth in xenograft models of lung adenocarcinoma, cervical cancer, and colorectal carcinoma [95,96,97]. Mechanistic studies implicated that Apoptozole-mediated tumor suppression was achieved by blocking interaction of HSP70 with APAF-1, which induced caspase-dependent apoptosis [96]. Park et al. reported that Apoptozole was also involved in promoting lysosome-mediated apoptosis and impairing autophagy in cancer cells [97]. Moreover, (-)-Epigallocatechin-3-gallate (EGCG), HSP70-36, Epoxysiderol, and Synthetic peptide P17 have also been found to exert anti-cancer effects in several studies [98,99,100,101,102]. Interestingly, EGCG and Epoxysiderol showed a selective binding affinity to HSP70, and they bind to HSPA5 and HSP70A1A, respectively [98,99,101]. Therefore, both of them might have the potential of being advanced in the clinical settings as anti-cancer drugs.

In addition to competitively binding to the ATP binding site, some other inhibitors can directly affect ATPase activity of HSP70 through interacting with a site outside of the ATP/ADP binding domain. YK5, a small molecule inhibitor which binds to an allosteric pocket HSP70, is one of the representatives. By specifically interacting with HSP70 isoforms, including HSPA1A/B and HSPA9, YK5 was identified to exhibit anti-tumor activity [103]. Rodina et al. found that YK5 induced the degradation of HER2, Raf1, and Akt kinases and promoted apoptosis in breast cancer cell lines [103]. However, the in vivo anti-tumor effect of YK5 still remains to be verified, and relevant research should be carried out as soon as possible. Recently, the interaction between HSP70 and Bim, a BH3-only member of the Bcl-2 family proteins, has been recognized as an effective target for cancer therapies [155]. Based on the established BH3 mimetics, two novel HSP70 inhibitors, S1g-2 and S1g-6, were developed to selectively disrupt HSP70–Bim protein–protein interaction (PPI) and inhibit the Bim-mediated activation of ATPase of HSP70 [104,106]. S1g-2 and S1g-6 significantly inhibited the growth of chronic myeloid leukemia in vitro and in vivo, and more importantly, S1g-2 exhibited an ever-growing ability to induce apoptosis and increase BCR-ABL independent TKI resistance in chronic myeloid leukemia cells [104,105,106]. Thus, S1g-2 and S1g-6 might belong to a completely new class of HSP70 inhibitor with promising anti-tumor activities in chronic myeloid leukemia.

MKT-077 is a cationic rhodacyanine dye analogue that targets an allosteric site which affects the binding of NEFs to HSPA8 and HSPA9 [110]. Research revealed that MKT-077 suppressed tumor growth through releasing wild-type p53 from the HSP70–p53 complex to rescue its transcriptional activity and clearing hyperphosphorylated tau in cells [107,108,109]. YM-01 and YM-08 belong to a new series of close derivatives of MKT-077. By disrupting the HSP70–BAG3 interaction, YM-01 modulated the activity of the transcription factors NF-κB, FoxM1, and Hif1α, the translation regulator HuR, and the cell cycle regulators p21 and survivin [111]. YM-08 can penetrate the blood–brain barrier, making it a potent inhibitor suitable for use in the central nervous system (CNS) malignancies or brain metastatic tumors. Unfortunately, compared with YM-01, which inhibited tumor growth of xenograft models of breast and melanoma, YM-08 had a reduced anti-tau activity and cytotoxicity in cancer cells [112,113]. Therefore, JG series of compounds related to YM-08 emerged [114,115,116,117,118]. Among them, JG-98 is the most extensively studied and therapeutically promising compound of the JG series in cancer treatments. Wu et al. reported that JG-98 showed greater potency than previous MKT-077 derivatives in terms of cytotoxicity against melanoma cells [114]. Moreover, JG-98 exhibits anti-tumor activity in breast and cervical cancer xenograft models through destabilizing FoxM1 and relieving suppression of downstream effectors, p21 and p27 [116].

MAL3-101 and its analog DMT3132 are small molecule inhibitors that affect allosteric communication associated with HSP70-JDP interaction [122,123]. Though both of them showed potent toxicity against cancers, DMT3132 exhibited stronger anti-proliferation activity than DMAL3-101 in breast cancer cells [95,121,123]. More importantly, when treated with MAL3-101, cancer cells became resistant to it by inducing autophagy through ATF4 signaling as well as endoplasmic reticulum-associated degradation (ERAD) [156,157]. Therefore, the potential combination therapy that synergizes with autophagy inhibitors is a new research direction in MAL3-101-based anti-cancer treatments.

##### SBD-Binding Inhibitors

The key roles of HSP70s in cancer-related molecular mechanisms are found to be mediated by PPIs between HSP70 and multiple proteins. SBD-binding inhibitors are specifically developed to disrupt these PPIs. The small molecule 2-phenylethyenesulfonamide (PES), also referred to as pifthrin-μ, is a selective inhibitor of stress-inducible HSP70 that not only promoted tumor cell death but also showed markedly less toxicity towards non-transformed cells [127,128]. It is cytotoxic against a variety of solid tumors, such as breast cancer, osteosarcoma, and pancreatic cancer, in addition to acute myeloid leukemia and acute lymphoblastic leukemia, regardless of p53 status or an elevated Bcl-xL protein level, and is caspase independent [102,124,126,127]. Additionally, PES can disrupt the HSP70/HSP90 chaperone system, resulting in the sequestration of several HSP90 client proteins into inactive, insoluble compartments [125]. In order to further enhance the medicinal properties of PES, Balaburski et al. developed a derivative of PES, 2-(3-chlorophenyl) ethynesulfonamide (PES-Cl), which showed comparable ability to bind to HSP70 but higher anti-tumor activity compared to PES. Through inhibiting autophagy and inducing programmed cell death, PES-Cl significantly prolonged the survival of Eμ-myc mice bearing pre-B cell lymphoma [129]. Another derivative of PES, Triphenyl (phenylethynyl) phosphonium (PET-16), and a derivative of PET-16, AP-4-139B, also exhibited strong anti-proliferation activity in cancer cells [128,130,131]. More importantly, AP-4-139B was identified to function effectively as a cancer vaccine. It released damage-associated molecular patterns (DAMPs) through disruption of mitochondrial function and increased immune cell recruitment into tumors [131]. Related structure–activity–relationship studies revealed that PET-16 specifically binds to the SBD of ADP-bound HSP70, and AP-4-139B binds to the same allosteric pocket as PET-16 [128,131]. All these data indicated that PES and its derivatives might be superior anti-cancer compounds.

Shortly after the discovery of PES, Hassan et al. indicated an HSP70-inhibiting role of the natural product novolactone, which interacts with an allosteric site in the SBD that affects the mobility of the lid and binding of JDPs in HSPA1A/B, HSPA5, and HSPA8 [132]. Although there has been no clear evidence that novolactone has anti-tumor activity, it can destabilize HER2 and EGFR in lung cancer cells [132]. Using a fluorescence polarization-based high-throughput screen, Ambrose et al. discovered the anti-infection agent hexachlorophene as an inhibitor of HSPA5. By leading to an unfolded protein response (UPR), hexachlorophene induced apoptosis and inhibited autophagy in colon cancer cell lines [133]. In addition, ADD70, a derivative of mitochondrial flavoprotein apoptosis inducing factor (AIF), and Acridizinium derivative 1, which disrupts HSP70 relocalization after heat shock, are two small molecule inhibitors that also bind to the SBD of HSP70. Subsequent studies revealed that they induced apoptosis in a broad range of tumor cells [134,135,136].

##### C-Terminal EEVD-Binding Inhibitors

A study published in 1995 showed that the extreme C-terminal four amino acids of HSP70 play an important role in modulating its ATPase activity, substrate binding, and interaction with HDJ-1 [158]. Subsequently, 15-Deoxyspergualin (DSG), which was proved to exert immunosuppressive function in numerous autoimmune diseases, binds specifically to this EEVD regulatory domain of HSPA8 [137]. This interaction between DSG and EEVD does not affect peptide binding and, on the contrary, may enhance the chaperone activity of HSP70 induced by ATP [137,140]. Similar to other NBD- or SBD-binding inhibitors, DSG was found to play an anti-cancer role in that it inhibits protein synthesis and induces apoptosis in lymphoma cells [138,139]. Still, more studies should be conducted to further corroborate this effect.

##### Other Inhibitors

With the development of cancer therapies targeting HSP70s, there are still quite a few promising anti-cancer inhibitors that directly or indirectly target HSP70 and have not clearly demonstrated the exact binding site on HSP70 or the effect on HSP70 activity. HA15, a compound of a series of thiazole benzenesulfonamides, was capable of inhibiting the ATPase activity of HSPA5 which is found in the endoplasmic reticulum (ER) and is a central regulator of the UPR [141]. HA-15 triggered apoptosis and induced autophagy in melanoma cells and prevented the growth of melanoma cells including those resistant to BRAF inhibitors in a mouse xenograft model [141]. Similarly, a natural product of the ritterazine-cephalostatin family, ritterostatin G_N_1_N_, showed selectivity for HSPA5 and exhibited a strong anti-cancer efficiency in melanoma [142]. N-amino-ethylamino derivative of colchicine (AEAC) was proved to have minimal cytotoxicity in glioma cells in normal conditions [159]. While under conditions of hypoxia, the antitumor effect of AEAC in glioblastoma was enhanced through disrupting PPI between HSP70 and GAPDH to promote the aggregation of oxidized GAPDH. Additionally, cells with high expression of GAPDH were more sensitive to AEAC than those with normal expression of GAPDH [143]. By disrupting the HSP70-caspase 3 complex, a derivative of benzodioxol (BT44) increased sensitivity of colon cancer and lymphoma cells to apoptosis [144].

Extracting compounds from natural plants is one of the commonly used methods to develop anti-cancer drugs. Triptolide, originally isolated from the Chinese herb *Tripterygium wilfordii*, and minnelide, a water-soluble pro-drug derivative of Triptolide, are both effective against multiple cancers, such as pancreatic cancer, colon cancer, neuroblastoma, osteosarcoma, malignant mesothelioma, and non-small cell lung cancer [145,146,147,148,149,150,151,152]. Mechanistically, this effect was probably mediated by the inhibition of HSP70 that triptolide and minnelide induced the binding of microRNA miR-142-3p to 3′UTR of HSP70 [149]. Therefore, triptolide and minnelide serve as a proof-of principle for indirectly targeting HSP70 as an anti-cancer therapy. When carefully examining these two drugs, triptolide has a higher specificity targeting tumor than minnelide because it was non-toxic to normal tissue cells. Unfortunately, triptolide is poorly soluble in water, limiting its clinical use [145,147]. Another natural compound flavonol quercetin was also identified to inhibit HSP70 expression and exhibited anti-proliferation activity in prostate cancer cells [92]. Based on the screening of a novel series of rhodacyanine-based HSP70 inhibitors, Tsai et al. revealed that compound 1 and compound 6 exhibited a high capacity for inhibiting the activity of HSP70′s chaperone and anti-proliferation activities against breast cancer cells [154]. A subsequent study conducted by them further revealed that treating xenograft models of triple negative breast cancer with compound 1 or compound 6 was also effective, and this was mediated by the down-regulation of β-catenin [153]. Nevertheless, it is important to focus on the binding mechanism of these compounds with HSP70 in future studies.

#### 4.1.2. HSP70 Inhibitors in Combination Therapy Mode

Although HSP70 inhibitors showed promising anti-tumor efficiency, the emergence of drug resistance limited their long-term benefit. Research revealed that endoplasmic reticulum-associated degradation (ERAD) and autophagy were activated in MAL3-101-resistant breast cancer, liver cancer, and rhabdomyosarcoma cells, and treatment with autophagy inhibitors restored their sensitivities to MAL3-101 [156,157]. Based on this, synergizing with other drugs seems to be a promising approach to enhance the anti-tumor effect of HSP70 inhibitors. S1g-6, an HSP70 inhibitor, plus Bcl-2/Mcl-1/Bcl-xl triple inhibitor S1 or FDA approved Bcl-2 inhibitor ABT-199 showed synergistic effect with S1g-6 in inducing tumor regression by inducing apoptosis in leukemia cells [160]. JG-98 plus HSP40 inhibitor C86 demonstrated the combinatorial activity in a CRPC xenograft model where HSP40 was found to be present in a multi-protein complex with full-length AR, ARv7, and HSP70 [161]. Additionally, combining HSP70 inhibitors, such as Apoptozole, VER-155008, PES, quercetin, or ADD70, with HSP90 inhibitors have also shown great therapeutic potential in numerous cancers, including muscle invasive bladder cancer, anaplastic thyroid carcinoma, melanoma, colorectal cancer, and acute myeloid leukemia [134,162,163,164,165,166,167]. The recommendation for this combination therapy modality was based on the results that the level of HSP70 was upregulated by HSP90 inhibitors and that dual inhibition of HSP70 and HSP90 could simultaneously disrupt the key signaling pathways in cancers [163,165,166].

A combination of HSP70 inhibitors plus chemotherapy or targeted therapy has also been widely explored in several studies. Platinum-based chemotherapy is the first-line standard treatment in many cancers. McKeon et al. discovered that compared with a single drug, PES plus oxaliplatin significantly improved anti-proliferation activity in colorectal cancer whereas PES plus cisplatin moderately improved the anti-cancer effect in prostate cancer [168]. Synergy of PES plus cisplatin was also reported in cervical cancer [169,170]. In breast cancer, EGCG increased etoposide-induced apoptosis in cells and suppressed the colony formation of cells treated with etoposide, indicative of the potential feasibility of the combination of these two drugs in treating breast cancer [99]. The administration of AEAC in combination with doxorubicin exerted a considerable therapeutic effect in glioma xenograft models [159]. In combined targeted therapy, sorafenib, a first-line targeted drug for advanced hepatocellular carcinoma, was reported to show a great anti-tumor effect when combined with HSP70 inhibitor triptolide [171]. PET-16 was found to reduce levels of mutant BRAF in melanoma as it synergized with the BRAF inhibitor PLX4032 by enhancing the durability of response to BRAF inhibition in vivo [172]. Additionally, a combination of HSP70 inhibitors and proteasome inhibitors suppressed tumor growth with a greater efficiency than single-agent treatments in melanoma and multiple myeloma [121,173,174].

Several studies revealed that malignant cells expressed higher levels of HSP70 than normal cells, and high expression of HSP70 induced resistance to radiotherapy through different mechanisms [175,176]. Thus, HSP70 inhibitors, such as triptolide which enhanced cellular radiosensitivity by inhibiting HSPA5 to trigger apoptosis and induce G_2_/M cell cycle arrest in nasopharyngeal carcinoma, can be used as radiosensitizers in radiation therapy [177]. Of note, the level of HSP70 was also upregulated in cancer cells treated with heat or light, leading to the low therapeutic efficiency in tumors [178,179]. Synergistic photothermal therapy (PTT), photodynamic therapy (PDT), or radiofrequency ablation (RFA) with HSP70 inhibitors, a combination treatment which has already shown improved efficiency than PTT, PDT, or RFA used alone in liver cancer, breast cancer, cervical cancer, and pancreatic cancer, offers an immediate translational potential in the management of numerous cancers [179,180,181,182,183].

### 4.2. HSP70 as an Adjuvant in Cancer Vaccine Therapies

Since Blachere and colleagues discovered that the HSP70–peptide complex leads to an antigen-specific CD8^+^ T cell response in 1997, a new research avenue has been opened for the use of HSP70 as an adjuvant in cancer vaccine therapies [122,123]. Numerous studies demonstrated the feasibility and effectiveness of HSP70 vaccines in combination with other substances, such as tumor-associated antigens (TAAs), tumor-specific antigens (TSAs), or proved the efficacy of tumor vaccines, as dendritic cell (DC), DNA, protein, or tumor cell lysate vaccines in anti-cancer therapies (Table 2). Some HSP70-based vaccines were designed to enhance the antigen-presenting capacity of DCs to T cells based on the results that HSP70 promoted DCs maturation, upregulation of co-stimulatory molecule, and cytokine secretion via interacting with DCs [184]. These vaccines that have been preliminarily proven effective in animal experiments include a tumor-derived autophagome (Dribble) vaccine conjugated with mycobacterial HSP70407–426 (M2) peptide, soluble form of B, and T lymphocyte attenuator (sBTLA) in combination with HSP70 vaccine where recombinant adeno-associated viral (AAV) vectors served as the gene delivery, DCs pulsed with recombinant fusion protein of CEA_576–669_ and HSP70-like protein 1 (HSP70L1), and DCs pulsed with tumor cell lysate which was pulsed with M2 peptide and OK-432 [185,186,187,188,189].

In the development of vaccines against HPV-associated tumors, such as cervical cancer, human papilloma virus (HPV)-encoded oncoproteins, particularly E7, are ideal target antigens since they can induce specific anti-tumor responses [213,214]. Fusion of HPV-16 E7 with HSP70 became a strategy to alleviate cellular immune responses to a DNA or protein vaccine in recent years [190,191,192,193,194,195,196,197]. TAAs and TSAs are also frequently used as vaccine components in combination with HSP70. Dickkopf-1 (DKK1), an ideal target for the immunotherapy of multiple myeloma, was conjugated to HSP70 as a DNA vaccine, and this constructed cancer vaccine was proved to have prophylactic and therapeutic anti-tumor effects through eliciting strong humoral and cellular immune responses in multiple myeloma [202]. Other TAAs and TSAs, such as alpha-fetoprotein (AFP), which is over-expressed in the majority of hepatocellular carcinoma, and prostate stem cell antigen (PSCA), which is associated with the development of prostate cancer, also showed great synergistic effect with HSP70 in multiple cancers [203,204,205,206,207,208,209,210,211]. A tumor cell lysate vaccine that was conjugated to diphtheria toxin (DT) and two tandem repeats of M2 peptide had protective anti-tumor immunity in a mouse breast tumor model [212]. Additionally, research regarding vaccine delivery approaches is ongoing [198,199,200,201]. Zhang et al. demonstrated that the nanoemulsion-encapsulated MAGE1-MAGE3-MAGEn/HSP70 fusion protein vaccine elicited stronger immune responses than those without nanoemulsion-encapsulation in hepatocellular carcinoma, suggesting that this novel nanoemulsion carrier induces potent anti-tumor immunity against the encapsulated antigens [198]. All these studies have demonstrated promising results, and the optimization of HSP70-based vaccines in terms of content, form, and delivery is still in progress.

### 4.3. HSP70-Based Cancer Therapies in Clinical Trials

Targeting HSP70 has been effective in preclinical studies; however, relevant clinical trials evaluating efficacy and safety of HSP70-based therapies have not yet made much progress to support their use in the treatment of cancer patients (Table 3). With the failure of phase I clinical trial of MKT-077 and phase II clinical trial of DSG, the future of clinical trials of HSP70 inhibitors in cancer therapies seems to be challenging [215]. On the contrary, several clinical trials of HSP70-based vaccines indicated that this therapeutic strategy seemed to be more promising as a novel cancer treatment. A HSP70 mRNA-transfected DC vaccine, a novel vaccination therapy comprising multi-HLA-binding HSP70/glypican-3 (GPC3) peptides and a novel adjuvant combination of hLAG-3Ig and Poly-ICLC, an autologous vaccine of leukocyte-derived HSP70-peptide complexes in conjunction with imatinib mesylate, and natural killer (NK) cells pulsed with the 14 amino acid sequence (aa(450–463)) TKDNNLLGRFELSG (TKD) of HSP70 and IL-2 were all found to be well tolerated among patients in phase I clinical trials [216,217,218,219]. Nevertheless, the assessment of targeting HSP70 in cancer therapies remains a major challenge and warrants further studies.

## 5. Conclusions and Future Directions

HSP70s are found to be over-expressed in many types of cancers, making it a potential target for cancer treatment. The main function of HSP70s is to serve as chaperones and collaborate with other co-chaperones to carry out house-keeping activities and to maintain protein stability. However, the effects of HSP70s on cancer cells are not quite dependent on their chaperone activities but rather on their abilities in regulating cancer cell signaling. Many signaling pathways can be directly regulate by HSP70s or indirectly by HSP70 clients. HSP70s mainly play a key role in promoting the most of common cancer pathways and various key proteins of other pathways. This is consistent with the fact that they are highly expressed in various types of cancers and enhance tumorigenesis. Although a large number of studies have shown that HSP70s promote these cancer-promoting pathways and related key proteins, few studies have elucidated whether and how HSP70s directly regulate these key proteins. Therefore, much more in-depth research on the molecular mechanism of HSP70s is warranted.

Anti-cancer therapy research targeting HSP70 has been carried out for more than 20 years. Numerous studies focused on HSP70 inhibitors reported great efficacies; however, there are still many obstacles in the transformation applications. One of the difficulties for developing HSP70 inhibitors is that HSP70 is ubiquitously expressed in the human body and has different isoforms. Further understanding the structure of HSP70, especially the SBD, and designing inhibitors based on this may be an effective way to solve the poor specificity of HSP70 inhibitors. Research on HSP70-based vaccines in cancer therapy is ongoing with a great potential. Several relevant phase I clinical trials have generated interesting preliminary data, making this approach more promising compared with HSP70 inhibitors. In addition to advancing to phase II–III clinical trials of the HSP70-based vaccines, another pressing issue that needs attention is how to validate the efficacy of these two therapeutic strategies targeting HSP70 in different types of cancers, given that HSP70 has a dual role in tumor progression. These two opposing roles of HSP70 may co-exist in the same tumor; therefore, research on the efficacy of targeting HSP70 is best evaluated in the immunocompetent animal models. Moreover, the expression level of HSP70 in tumor cells and the immunogenicity of this tumor may help to choose whether to preferentially verify the efficacy of HSP70 inhibitors or HSP70-based vaccines.

It is important to note that the critical role of HSP70 in cancer progression makes it alternative target protein for patients who failed in treatment of chemotherapy, targeted therapy, or immunotherapy. The widespread expression of HSP70 in tumors makes targeting HSP70 in cancer treatment full of promise. More importantly, the safety of this therapeutic strategy obtained by the current research is also reliable. In the next stage of research, further elucidation of HSP70s’ functions in cancers, such as HSP70 binding sites, substrate proteins, and corresponding signaling pathways will allow us to develop and test HSP70-based anti-cancer therapies with greater clinical efficacies.

## Figures and Tables

**Table 1:** Preclinical studies of HSP70 inhibitors in cancer therapies.

Binding Sites	Order	Inhibitor Name	Chemical Structures	Effects on HSP70 Activity	Cancer Types	Effects on Cancers	Mechanisms	Refs
NBD	1	VER-155008	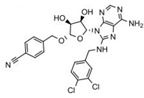	Inhibits ATPase activity of HSP70 (all isoforms) through interacting with the ATP binding site	Vismodegib-resistant basal cell carcinoma, colon cancer, mesothelioma, pheochromocytoma, castration-resistant prostate cancer, anaplastic thyroid carcinoma, breast cancer	Inhibits tumor cell proliferation, invasion, and migration in vitro; induces cell cycle arrest, apoptosis, and autophagy in vitro; inhibits tumor growth in xenograft models	Decreases the expression of the downstream Hedgehog target gene Gli1; down-regulates phosphorylation of the PI3K/AKT/mTOR and MEK/ERK signaling pathways	[59,86,87,88,89,90,91,92,93,94]
	2	Apoptozole	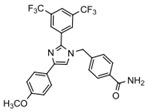	Inhibits ATPase activity of HSP70 through interacting with the ATP binding site	A broad spectrum of cancer cells	Inhibits tumor cell proliferation and migration in vitro; induces cell apoptosis in vitro; inhibits tumor growth in xenograft models	Induces caspase-dependent apoptosis by blocking interaction of HSP70 with APAF-1; promotes lysosome-mediated apoptosis and impairs autophagy	[95,96,97]
	3	EGCG	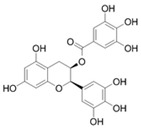	Inhibits ATPase activity of HSP70 (HSPA5) through interacting with the ATP binding site	Breast cancer, colon cancer	Inhibits tumor cell proliferation and induce apoptosis in vitro; inhibits tumor growth in xenograft models	Induces caspase-dependent apoptosis by blocking interaction of HSPA5 with caspase-7	[98,99]
	4	HSP70-36	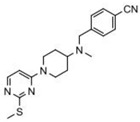	Inhibits ATPase activity of HSP70 through interacting with the ATP binding site	lapatinib-resistant breast cancer	Inhibits tumor cell proliferation in vitro		[100]
	5	Epoxysiderol	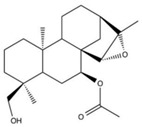	Inhibits ATPase activity of HSP70 (HSP70A1A) through interacting with the ATP binding site	Cervical cancer	Induces cell cycle arrest and apoptosis in vitro	Decreases the expression of pAkt, pERK2, JNK1 and p38	[101]
	6	Synthetic peptide P17		Inhibits ATPase activity of HSP70 through interacting with the ATP binding site	Melanoma	Inhibits tumor growth in vivo	Triggers an antitumor immune response	[102]
	7	YK5	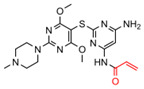	Inhibits ATPase activity of HSP70 (HSPA1A/B, A9) through interacting with a site outside the ATP binding domain	Breast cancer	Induces tumor cell apoptosis in vitro	Induces the degradation of HER2, Raf1, and Akt kinases	[103]
	8	S1g-2	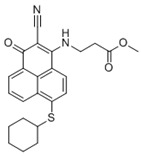	Inhibits ATPase activity of HSP70 through disrupting HSP70-Bim PPI	TKI-resistant chronic myeloid leukemia	Inhibits tumor cell proliferation and induces apoptosis in vitro; inhibits tumor growth in vivo	Down-regulates the level of key oncoclient proteins including AKT, Raf-1, eIF4E, and RPS16	[104,105]
	9	S1g-6		Inhibits ATPase activity of HSP70 through disrupting HSP70-Bim PPI	Chronic myeloid leukemia	Induces tumor cell apoptosis in vitro	Decreases the expression and phosphorylation levels of AKT and Raf-1	[106]
	10	MKT-077	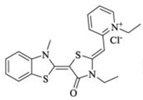	Inhibits HSP70 (HSPA8, A9)-NEFs complex	Breast cancer, ovarian cancer, endometrial cancer, colon cancer, non-small cell lung cancer, cervical cancer, osteosarcoma, and melanoma	Inhibits tumor cell proliferation and induces senescence in vitro	Releases wild-type p53 from HSP70-p53 complex; reduces tau levels	[107,108,109,110]
	11	YM-01 (MKT-077 derivative)	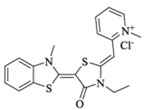	Inhibits HSP70-NEFs complex	Breast cancer, cervical cancer, melanoma	Inhibits tumor growth in xenograft models	Affects the activity of several transcription factors NF-κB, FoxM1, and Hif1α, translation regulator HuR, and cell cycle regulators p21 and survivin	[111,112]
	12	YM-08 (MKT-077 derivative)	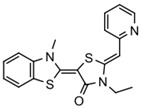	Inhibits HSP70 (HSPA8, A9)-NEFs complex	Breast cancer, cervical cancer	Has a low cytotoxicity to tumor cell viability in vitro	BBB penetrant; reduces tau levels	[113]
	13	JG-98 (MKT-077 derivative)	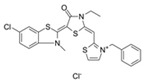	Inhibits HSP70 (HSPA1A, A5, A8, A9)-NEFs complex	B-Raf^V600E^ melanoma, breast cancer, cervical cancer, skin cancer, ovarian cancer, bone marrow cancer	Inhibits tumor cell proliferation and induce apoptosis in vitro; inhibits tumor growth in xenograft models	Destabilizes FoxM1 and relieved suppression of downstream effectors, p21 and p27; destabilizes Akt1 and Raf1	[114,115,116]
	14	JG2-38 (MKT-077 derivative)	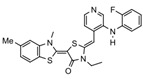	Inhibits HSP70 (HSPA1A)-NEFs complex	Breast cancer, prostate cancer	Inhibits tumor cell proliferation in vitro		[117]
	15	JG-231 (MKT-077 derivative)	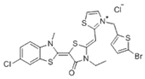	Inhibits HSP70 (HSPA9)-NEFs complex	B-Raf^V600E^ melanoma, breast cancer	Inhibits tumor growth in xenograft models	Increases mitochondrial permeability through deregulating MEK-ERK activity	[114,118]
	16	HS-72	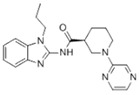	Inhibits HSP70 (HSPA1A/B)-NEFs complex	Breast cancer, prostate cancer	Inhibits tumor growth in vitro and in vivo		[119]
	17	MAL3-101	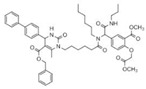	Inhibits HSP70-JDP complex	Merkel cell carcinoma, multiple myeloma	Inhibits tumor cell proliferation and induce apoptosis in vitro; inhibits tumor growth in xenograft models		[120,121,122]
	18	DMT3132 (MAL3-101 analog)	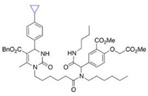	Inhibits HSP70-JDP complex	Breast cancer	Inhibits tumor cell proliferation in vitro		[123]
SBD	19	PES	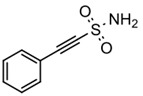	Inhibits HSP70 (HSPA1A/B)-client interaction	NSCLC, breast cancer, Osteosarcoma, pancreatic cancer, Myc-Induced Lymphoma, acute myeloid leukemia, acute lymphoblastic leukemia	Inhibits tumor cell proliferation and migration in vitro; induces apoptosis and cell cycle arrest in vitro; inhibits tumor growth in xenograft models;	Down-regulates phosphorylation of AKT and ERK; causes dysfunctional autophagy and altered lysosomal function	[124,125,126,127,128]
	20	PES-Cl (PES derivative)	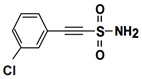	Inhibits HSP70-client interaction	B-cell lymphoma, melanoma	inhibits tumor cell proliferation and autophagy in vitro; induces cell cycle arrest and genomic instability in cancer cells; inhibits tumor growth in xenograft models		[129]
	21	PET-16 (PES derivative)	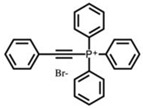	Inhibits HSP70-client interaction	Multiple myeloma, melanoma	Inhibits tumor cell proliferation and induces apoptosis in vitro		[128,130]
	22	AP-4-139B (PET-16 derivative)	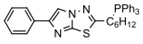	Inhibits HSP70-client interaction	Colorectal cancer	Inhibits tumor growth in vitro and in vivo	Releases DAMPs through disruption of mitochondrial function and increases immune cell recruitment into tumors	[131]
	23	Novolactone	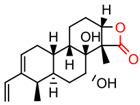	Inhibits JDP-dependent ATPase activity and the allosteric control mechanism between the SBD and the NBD of HSP70 (HSPA1A/B, A5, A8)	Lung cancer		Destabilizes HER2 and EGFR in cancer cells	[132]
	24	Hexachlorophene	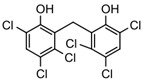	Inhibits HSP70 (HSPA5)-client interaction	Colon cancer	Induces tumor cell apoptosis and inhibits autophagy in vitro		[133]
	25	ADD70		Unknown	Colorectal cancer, melanoma, breast cancer, cervical cancer, leukemia	Increases sensitivity of tumor cells to apoptosis in vitro; inhibits tumor growth in vivo	Improves cytotoxic activity of CD8^+^ tumor-infiltrating T cells	[134,135]
	26	Acridizinium derivative 1	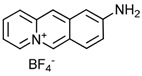	Inhibits HSP70 relocalization after heat shock	Cervical cancer	Induces tumor cell apoptosis in vitro		[136]
C-terminal EEVD motif D	27	DSG	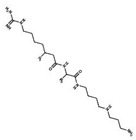	Unknown	Lymphoma	Inhibits protein synthesis and induces apoptosis in vitro; inhibits tumor growth in vivo	Inhibits activation of p70S6K and Akt	[137,138,139,140]
Unknown	28	HA15	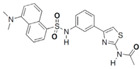	Inhibits ATPase activity of HSP70 (HSPA5)	Melanoma, prostate cancer, breast cancer, colon cancer, pancreatic cancer, glioma, cervical cancer	Induces tumor cell apoptosis and inhibits autophagy in vitro; inhibits tumor growth in xenograft models	Induces ER stress	[141]
	29	Ritterostatin G_N_1_N_	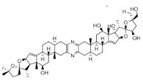	Inhibits HSP70 (HSPA5) activity	Melanoma, colon cancer	Inhibits tumor cell proliferation in vitro	Induces ER stress and UPR	[142]
	30	AEAC		Disrupts HSP70-oxidized GAPDH complex	Glioblastoma	Inhibits tumor growth in vitro and in vivo	Promotes the aggregation of oxidized GAPDH and increases sensitivity of cells to hypoxic stress	[143]
	31	BT-44	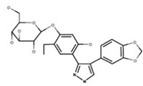	Disrupts HSP70-Caspase-3 complex	Colon cancer, lymphoma	Increases sensitivity of tumor cells to apoptosis in vitro		[144]
	32	Triptolide	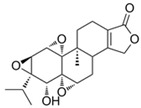	Reduces the expression level of HSP70	Pancreatic cancer, colon cancer, neuroblastoma, osteosarcoma, malignant mesothelioma, non-small cell lung cancer	Inhibits tumor cell proliferation in vitro; induces apoptosis and cell cycle arrest in vitro; inhibits tumor growth and decreases local-regional tumor spread in vivo	Down-regulates E2F activity by modulating events downstream of DNA binding; downregulates the levels of pro-survival proteins such as cMYC and survivin and targets NF-κB pathway	[145,146,147,148,149,150,151]
	33	Minnelide (Triptolide derivative)	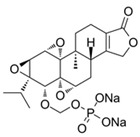	Reduces the expression level of HSP70	Colon cancer, osteosarcoma, malignant mesothelioma, non-small cell lung cancer	Inhibits the tumor growth in the xenograft and metastasis model	Downregulates the levels of pro-survival proteins such as cMYC and survivin and targets NF-κB pathway	[146,147,149,150,151,152]
	34	Quercetin	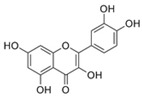	Reduces the expression level of HSP70	Prostate cancer	Inhibits tumor cell proliferation and induces apoptosis in vitro		[92]
	35	Rhodacyanine-based compound 1	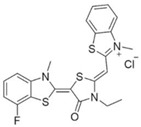	Inhibits the chaperone activity of HSP70	Triple negative breast cancer	Inhibits tumor growth in vitro and in vivo	Down-regulates β-catenin	[153,154]
	36	Rhodacyanine-based compound 6	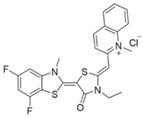	Inhibits the chaperone activity of HSP70	Triple negative breast cancer	Inhibits tumor growth in vitro and in vivo	Down-regulates β-catenin	[153,154]

**Table 2:** Preclinical studies of HSP70-based vaccines in cancer therapies.

Order	Vaccines	Vaccine Types	Cancer Types	Administration Methods	Immunotherapy Effects	Immune Mechanisms	Refs
1	E7-HSP70 (optimizedE7/mHSP70, modifiedE7/mtHSP70, optimizedE7/hHSP70, sigmE7/mtHSP70, modifiedE7/HSP70)	DNA vaccine, protein vaccine	Cervical cancer, lung metastatic melanoma	Subcutaneous injection, intramuscular injection	Prophylactic and therapeutic antitumor effects	Cellular immune response	[190,191,192,193,194,195,196,197]
2	MAGE-HSP70 (MAGE1-MAGE3-MAGEn/HSP70, TL-MAGE1-HSP70/SEA, MAGE1/HSP70, MAGE1-HSP70/SEA)	Nanoemulsion-encapsulated protein vaccine, recombinant protein vaccine	Hepatocellular carcinoma, melanoma,	Intraperitoneal injection, oral, subcutaneous injection	Prophylactic and therapeutic antitumor effects	Humoral and cellular immune responses	[198,199,200,201]
3	hDKK1-hHSP70	DNA vaccine	Multiple myeloma	Intramuscular injection	Prophylactic and therapeutic antitumor effects	Humoral and cellular immune responses	[202]
4	AFP-HSP70	DNA vaccine, protein vaccine	Hepatocellular carcinoma	Intramuscular injection	Therapeutic antitumor effect	Cellular immune response	[203,204]
5	PSCA-HSP70	Protein vaccine, DNA vacine	Prostate cancer	Subcutaneous injection, intramuscular injection	Therapeutic antitumor effect	Humoral and cellular immune responses	[205,206]
6	Mela-HSP70	Protein vaccine	Melanoma	Subcutaneous injection	Prophylactic antitumor effect	Cellular immune response	[207]
7	OVA_257–264_-HSP70	DNA vaccine	Lymphoma	Intradermal injection	Prophylactic antitumor effect	Cellular immune response	[208]
8	A20Id-mycHSP70	Protein vaccine	Lymphoma	Intraperitoneal injection	Prophylactic antitumor effect	Humoral and cellular immune responses	[209]
9	LV-TRP2-HSP70	Lentiviral vector vaccine	Melanoma, breast cancer, glioblastoma	Subcutaneous injection	Therapeutic antitumor effect	Cellular immune response	[210]
10	DRibble vaccine+mtHSP70_407–426_ (M2)	Conjugated vaccine	Lung cancer	Subcutaneous injection	Therapeutic antitumor effect	Cellular immune response	[185]
11	HUVEC vaccine+mtHSP70_407–426_ (M2)	Conjugated vaccine	Hepatocellular carcinoma	Subcutaneous injection	Prophylactic and therapeutic antitumor effects	Humoral and cellular immune responses	[211]
12	BTLA vaccine+HSP70 vaccine (AAV-sBTLA vaccine+HSP70 vaccine, psBTLA vaccine+HSP70 vaccine)	Recombinant adenovirus vector vaccine+protein vaccine, DNA vaccine+protein vaccine	Lung metastatic melanoma, cervical cancer	Tail vein injection, intramuscular injection	Prophylactic and therapeutic antitumor effects	Cellular immune response	[186,187]
13	DT-TCL-mtHSP70_407–426_ (M2)	Tumor cell lysate vaccine	Breast cancer	Subcutaneous injection	Prophylactic antitumor effect	Humoral and cellular immune responses	[212]
14	DCs pulsed with CEA_576–669_-HSP70L1	DC vaccine	Colon cancer	Intraperitoneal injection	Therapeutic antitumor effect	Cellular immune response	[188]
15	DCs pulsed with tumor cell lysate pulsed with mtHSP70_407–426_ (M2) and OK-432	DC vaccine	Hepatocellular carcinoma	Subcutaneous injection	Therapeutic antitumor effect	Cellular immune response	[189]

hHSP70, human HSP70; mHSP70, murine HSP70; mtHSP70, mycobacterium tuberculosis HSP70; mycHSP70, mycobacterial HSP70; MAGE, melanoma-associated antigen gene; TL, tomato lectin; SEA, staphylococcal enterotoxins A; DKK1, Dickkopf-1; AFP, alpha-fetoprotein; PSCA, prostate stem-cell antigen; OVA, ovalbumin; LV, lentiviral vector; TRP2, tyrosinase-related protein-2; Dribble, tumor-derived autophagome; HUVEC, human umbilical vein endothelial cell; BTLA, B and T lymphocyte attenuator; AAV, adeno-associated virus; DT, diphtheria toxin; TCL, tumor cell lysates; DC, dendritic cell; CEA, carcinoembryonic antigen; HSP70L1, HSP70-like protein 1.

**Table 3:** HSP70-based cancer therapies in clinical trials.

Therapy Type	NCT Number	Drug Name or Vaccine Component	Inclusion Criteria	Study Type	Clinical Phase	Recruitment Status	Publication
HSP70 inhibitors	NCT01927965	Minnelide	Advanced gastrointestinal tumors	Non-randomized/one-arm	Phase I	Completed	
	NCT04896073	Minnelide	Advanced refractory adenosquamous carcinoma of the pancreas	Non-randomized/one-arm	Phase II	Recruiting	[220]
HSP70-based vaccines	NCT00027144	Autologous tumor-derived HSP70 protein vaccine	Chronic myeloid leukemia	Non-randomized/one-arm	Phase I	Completed	
	NCT00030303	Autologous PMNCs-derived HSP70 protein vaccine	Chronic myeloid leukemia	Non-randomized/one-arm	Phase I	Completed	[219]
	NCT00005633	Tyrosinase and gp100 peptides fused with OVA BiP peptide and recombinant HSP70 protein	Stage III or stage IV melanoma	Non-randomized/one-arm	Phase I	Completed	
	NCT05059821	Autologous activated monocytes with autologous tumor-derived HSP70 protein vaccine	Hepatocellular carcinoma who developed recurrence after surgical resection and refractory to the available institutional standard of care lines of treatment	Non-randomized/one-arm	Phase I	Recruiting	

## Data Availability

Not applicable.

## Supplementary Materials

The following supporting information can be downloaded at: https://www.mdpi.com/article/10.3390/biom13040601/s1, Table S1: HSP70 family in human.

## References

[1] Ritossa F. (1962). A new puffing pattern induced by temperature shock and DNP in drosophila. Cell. Mol. Life Sci..

[2] Jäättelä M., Saksela K., Saksela E. (1989). Heat shock protects WEHI-164 target cells from the cytolysis by tumor necrosis factors alpha and beta. Eur. J. Immunol..

[3] Petersen N.S., Mitchell H.K. (1981). Recovery of protein synthesis after heat shock: Prior heat treatment affects the ability of cells to translate mRNA. Proc. Natl. Acad. Sci. USA.

[4] Sapareto S.A., Hopwood L.E., Dewey W.C., Raju M.R., Gray J.W. (1978). Effects of hyperthermia on survival and progression of Chinese hamster ovary cells. Cancer Res..

[5] Henle K.J., Karamuz J.E., Leeper D.B. (1978). Induction of thermotolerance in Chinese hamster ovary cells by high (45 degrees) or low (40 degrees) hyperthermia. Cancer Res..

[6] Gerner E.W., Boone R., Connor W.G., Hicks J.A., Boone M.L. (1976). A transient thermotolerant survival response produced by single thermal doses in HeLa cells. Cancer Res..

[7] Ritossa F. (1996). Discovery of the heat shock response. Cell Stress Chaperones.

[8] Kampinga H.H., Hageman J., Vos M.J., Kubota H., Tanguay R.M., Bruford E.A., Cheetham M.E., Chen B., Hightower L.E. (2009). Guidelines for the nomenclature of the human heat shock proteins. Cell Stress Chaperones.

[9] Rosenzweig R., Nillegoda N.B., Mayer M.P., Bukau B. (2019). The Hsp70 chaperone network. Nat. Reviews. Mol. Cell Biol..

[10] Albakova Z., Armeev G.A., Kanevskiy L.M., Kovalenko E.I., Sapozhnikov A.M. (2020). HSP70 Multi-Functionality in Cancer. Cells.

[11] Murphy M.E. (2013). The HSP70 family and cancer. Carcinogenesis.

[12] Sherman M.Y., Gabai V.L. (2014). Hsp70 in cancer: Back to the future. Oncogene.

[13] Ciocca D.R., Calderwood S.K. (2005). Heat shock proteins in cancer: Diagnostic, prognostic, predictive, and treatment implications. Cell Stress Chaperones.

[14] Calderwood S.K., Khaleque M.A., Sawyer D.B., Ciocca D.R. (2006). Heat shock proteins in cancer: Chaperones of tumorigenesis. Trends Biochem. Sci..

[15] Brondani Da Rocha A., Regner A., Grivicich I., Pretto Schunemann D., Diel C., Kovaleski G., Brunetto De Farias C., Mondadori E., Almeida L., Braga Filho A. (2004). Radioresistance is associated to increased Hsp70 content in human glioblastoma cell lines. Int. J. Oncol..

[16] Yang Z., Zhuang L., Szatmary P., Wen L., Sun H., Lu Y., Xu Q., Chen X. (2015). Upregulation of heat shock proteins (HSPA12A, HSP90B1, HSPA4, HSPA5 and HSPA6) in tumour tissues is associated with poor outcomes from HBV-related early-stage hepatocellular carcinoma. Int. J. Med. Sci..

[17] Jin H., Ji M., Chen L., Liu Q., Che S., Xu M., Lin Z. (2016). The clinicopathological significance of Mortalin overexpression in invasive ductal carcinoma of breast. J. Exp. Clin. Cancer Res. CR.

[18] Radons J. (2016). The human HSP70 family of chaperones: Where do we stand?. Cell Stress Chaperones.

[19] Hunt C., Morimoto R.I. (1985). Conserved features of eukaryotic hsp70 genes revealed by comparison with the nucleotide sequence of human hsp70. Proc. Natl. Acad. Sci. USA.

[20] Gupta R.S., Singh B. (1994). Phylogenetic analysis of 70 kD heat shock protein sequences suggests a chimeric origin for the eukaryotic cell nucleus. Curr. Biol. CB.

[21] Flaherty K.M., DeLuca-Flaherty C., McKay D.B. (1990). Three-dimensional structure of the ATPase fragment of a 70K heat-shock cognate protein. Nature.

[22] Arakawa A., Handa N., Shirouzu M., Yokoyama S. (2011). Biochemical and structural studies on the high affinity of Hsp70 for ADP. Protein Sci. A Publ. Protein Soc..

[23] Vogel M., Mayer M.P., Bukau B. (2006). Allosteric regulation of Hsp70 chaperones involves a conserved interdomain linker. J. Biol. Chem..

[24] Zhu X., Zhao X., Burkholder W.F., Gragerov A., Ogata C.M., Gottesman M.E., Hendrickson W.A. (1996). Structural analysis of substrate binding by the molecular chaperone DnaK. Science.

[25] Kityk R., Vogel M., Schlecht R., Bukau B., Mayer M.P. (2015). Pathways of allosteric regulation in Hsp70 chaperones. Nat. Commun..

[26] Zhuravleva A., Clerico E.M., Gierasch L.M. (2012). An interdomain energetic tug-of-war creates the allosterically active state in Hsp70 molecular chaperones. Cell.

[27] Mayer M.P., Schröder H., Rüdiger S., Paal K., Laufen T., Bukau B. (2000). Multistep mechanism of substrate binding determines chaperone activity of Hsp70. Nat. Struct. Biol..

[28] Zuiderweg E.R., Hightower L.E., Gestwicki J.E. (2017). The remarkable multivalency of the Hsp70 chaperones. Cell Stress Chaperones.

[29] Scheufler C., Brinker A., Bourenkov G., Pegoraro S., Moroder L., Bartunik H., Hartl F.U., Moarefi I. (2000). Structure of TPR domain-peptide complexes: Critical elements in the assembly of the Hsp70-Hsp90 multichaperone machine. Cell.

[30] Odunuga O.O., Hornby J.A., Bies C., Zimmermann R., Pugh D.J., Blatch G.L. (2003). Tetratricopeptide repeat motif-mediated Hsc70-mSTI1 interaction. Molecular characterization of the critical contacts for successful binding and specificity. J. Biol. Chem..

[31] Laufen T., Mayer M.P., Beisel C., Klostermeier D., Mogk A., Reinstein J., Bukau B. (1999). Mechanism of regulation of hsp70 chaperones by DnaJ cochaperones. Proc. Natl. Acad. Sci. USA.

[32] Mayer M.P., Bukau B. (2005). Hsp70 chaperones: Cellular functions and molecular mechanism. Cell. Mol. Life Sci. CMLS.

[33] Evdonin A., Kinev A., Tsupkina N., Guerriero V., Raynes D.A., Medvedeva N. (2009). Extracellular HspBP1 and Hsp72 synergistically activate epidermal growth factor receptor. Biol. Cell.

[34] Li R., Yanjiao G., Wubin H., Yue W., Jianhua H., Huachuan Z., Rongjian S., Zhidong L. (2017). Secreted GRP78 activates EGFR-SRC-STAT3 signaling and confers the resistance to sorafeinib in HCC cells. Oncotarget.

[35] Yin Y., Chen C., Chen J., Zhan R., Zhang Q., Xu X., Li D., Li M. (2017). Cell surface GRP78 facilitates hepatoma cells proliferation and migration by activating IGF-IR. Cell. Signal..

[36] Bonvini P., Zorzi E., Basso G., Rosolen A. (2007). Bortezomib-mediated 26S proteasome inhibition causes cell-cycle arrest and induces apoptosis in CD-30+ anaplastic large cell lymphoma. Leukemia.

[37] Bonvini P., Zorzi E., Mussolin L., Pillon M., Romualdi C., Peron M., d’Amore E.S., Lamant L., Rosolen A. (2012). Consequences of heat shock protein 72 (Hsp72) expression and activity on stress-induced apoptosis in CD30+ NPM-ALK+ anaplastic large-cell lymphomas. Leukemia.

[38] Meng L., Hunt C., Yaglom J.A., Gabai V.L., Sherman M.Y. (2011). Heat shock protein Hsp72 plays an essential role in Her2-induced mammary tumorigenesis. Oncogene.

[39] Shen J., Ha D.P., Zhu G., Rangel D.F., Kobielak A., Gill P.S., Groshen S., Dubeau L., Lee A.S. (2017). GRP78 haploinsufficiency suppresses acinar-to-ductal metaplasia, signaling, and mutant Kras-driven pancreatic tumorigenesis in mice. Proc. Natl. Acad. Sci. USA.

[40] Wu P.K., Hong S.K., Starenki D., Oshima K., Shao H., Gestwicki J.E., Tsai S., Park J.I. (2020). Mortalin/HSPA9 targeting selectively induces KRAS tumor cell death by perturbing mitochondrial membrane permeability. Oncogene.

[41] Rangel D.F., Dubeau L., Park R., Chan P., Ha D.P., Pulido M.A., Mullen D.J., Vorobyova I., Zhou B., Borok Z. (2021). Endoplasmic reticulum chaperone GRP78/BiP is critical for mutant Kras-driven lung tumorigenesis. Oncogene.

[42] Ha D.P., Huang B., Wang H., Rangel D.F., Van Krieken R., Liu Z., Samanta S., Neamati N., Lee A.S. (2022). Targeting GRP78 suppresses oncogenic KRAS protein expression and reduces viability of cancer cells bearing various KRAS mutations. Neoplasia.

[43] Song J., Takeda M., Morimoto R.I. (2001). Bag1-Hsp70 mediates a physiological stress signalling pathway that regulates Raf-1/ERK and cell growth. Nat. Cell Biol..

[44] Wu P.K., Hong S.K., Park J.I. (2017). Steady-State Levels of Phosphorylated Mitogen-Activated Protein Kinase Kinase 1/2 Determined by Mortalin/HSPA9 and Protein Phosphatase 1 Alpha in KRAS and BRAF Tumor Cells. Mol. Cell. Biol..

[45] Kelber J.A., Panopoulos A.D., Shani G., Booker E.C., Belmonte J.C., Vale W.W., Gray P.C. (2009). Blockade of Cripto binding to cell surface GRP78 inhibits oncogenic Cripto signaling via MAPK/PI3K and Smad2/3 pathways. Oncogene.

[46] Misra U.K., Pizzo S.V. (2010). Ligation of cell surface GRP78 with antibody directed against the COOH-terminal domain of GRP78 suppresses Ras/MAPK and PI 3-kinase/AKT signaling while promoting caspase activation in human prostate cancer cells. Cancer Biol. Ther..

[47] Lu M.C., Lai N.S., Yin W.Y., Yu H.C., Huang H.B., Tung C.H., Huang K.Y., Yu C.L. (2013). Anti-citrullinated protein antibodies activated ERK1/2 and JNK mitogen-activated protein kinases via binding to surface-expressed citrullinated GRP78 on mononuclear cells. J. Clin. Immunol..

[48] Hu Y., Yang L., Yang Y., Han Y., Wang Y., Liu W., Zuo J. (2016). Oncogenic role of mortalin contributes to ovarian tumorigenesis by activating the MAPK-ERK pathway. J. Cell. Mol. Med..

[49] Somensi N., Brum P.O., de Miranda Ramos V., Gasparotto J., Zanotto-Filho A., Rostirolla D.C., da Silva Morrone M., Moreira J.C.F., Pens Gelain D. (2017). Extracellular HSP70 Activates ERK1/2, NF-kB and Pro-Inflammatory Gene Transcription Through Binding with RAGE in A549 Human Lung Cancer Cells. Cell. Physiol. Biochem..

[50] Liu Q., Li Y., Zhou L., Li Y., Xu P., Liu X., Lv Q., Li J., Guo H., Cai H. (2018). GRP78 Promotes Neural Stem Cell Antiapoptosis and Survival in Response to Oxygen-Glucose Deprivation (OGD)/Reoxygenation through PI3K/Akt, ERK1/2, and NF-κB/p65 Pathways. Oxidative Med. Cell. Longev..

[51] Cao L., Yuan X., Bao F., Lv W., He Z., Tang J., Han J., Hu J. (2019). Downregulation of HSPA2 inhibits proliferation via ERK1/2 pathway and endoplasmic reticular stress in lung adenocarcinoma. Ann. Transl. Med..

[52] Wu P.K., Hong S.K., Park J.I. (2021). Mortalin depletion induces MEK/ERK-dependent and ANT/CypD-mediated death in vemurafenib-resistant B-Raf(V600E) melanoma cells. Cancer Lett..

[53] Liu P., Cheng H., Roberts T.M., Zhao J.J. (2009). Targeting the phosphoinositide 3-kinase pathway in cancer. Nat. Rev. Drug Discov..

[54] Chen W., Liu X., Yuan S., Qiao T. (2018). HSPA12B overexpression induces cisplatin resistance in non-small-cell lung cancer by regulating the PI3K/Akt/NF-κB signaling pathway. Oncol. Lett..

[55] Fu R., Yang P., Wu H.L., Li Z.W., Li Z.Y. (2014). GRP78 secreted by colon cancer cells facilitates cell proliferation via PI3K/Akt signaling. Asian Pac. J. Cancer Prev. APJCP.

[56] Liu H., Li Z., Li Q., Jia C., Zhang N., Qu Y., Hu D. (2021). HSP70 inhibition suppressed glioma cell viability during hypoxia/reoxygenation by inhibiting the ERK1/2 and PI3K/AKT signaling pathways. J. Bioenerg. Biomembr..

[57] Liu R., Li X., Gao W., Zhou Y., Wey S., Mitra S.K., Krasnoperov V., Dong D., Liu S., Li D. (2013). Monoclonal antibody against cell surface GRP78 as a novel agent in suppressing PI3K/AKT signaling, tumor growth, and metastasis. Clin. Cancer Res..

[58] Zhang Y., Tseng C.C., Tsai Y.L., Fu X., Schiff R., Lee A.S. (2013). Cancer cells resistant to therapy promote cell surface relocalization of GRP78 which complexes with PI3K and enhances PI(3,4,5)P3 production. PloS ONE.

[59] Xu F., Lin D., Jiang W., Meng L., Xu Y., Wang C., Wang X., He H., Xu D., Zhu Y. (2019). HSP70 inhibitor VER155008 suppresses pheochromocytoma cell and xenograft growth by inhibition of PI3K/AKT/mTOR and MEK/ERK pathways. Int. J. Clin. Exp. Pathol..

[60] Fu Y., Wey S., Wang M., Ye R., Liao C.P., Roy-Burman P., Lee A.S. (2008). Pten null prostate tumorigenesis and AKT activation are blocked by targeted knockout of ER chaperone GRP78/BiP in prostate epithelium. Proc. Natl. Acad. Sci. USA.

[61] Wey S., Luo B., Tseng C.C., Ni M., Zhou H., Fu Y., Bhojwani D., Carroll W.L., Lee A.S. (2012). Inducible knockout of GRP78/BiP in the hematopoietic system suppresses Pten-null leukemogenesis and AKT oncogenic signaling. Blood.

[62] Chen W.T., Zhu G., Pfaffenbach K., Kanel G., Stiles B., Lee A.S. (2014). GRP78 as a regulator of liver steatosis and cancer progression mediated by loss of the tumor suppressor PTEN. Oncogene.

[63] Yang L., Guo W., Zhang Q., Li H., Liu X., Yang Y., Zuo J., Liu W. (2011). Crosstalk between Raf/MEK/ERK and PI3K/AKT in suppression of Bax conformational change by Grp75 under glucose deprivation conditions. J. Mol. Biol..

[64] Kim H.J., Kim S.Y., Kim D.H., Park J.S., Jeong S.H., Choi Y.W., Kim C.H. (2021). Crosstalk between HSPA5 arginylation and sequential ubiquitination leads to AKT degradation through autophagy flux. Autophagy.

[65] Chen S., Wu J., Jiao K., Wu Q., Ma J., Chen D., Kang J., Zhao G., Shi Y., Fan D. (2018). MicroRNA-495-3p inhibits multidrug resistance by modulating autophagy through GRP78/mTOR axis in gastric cancer. Cell Death Dis..

[66] Ryu H.H., Ha S.H. (2020). HSP70 interacts with Rheb, inhibiting mTORC1 signaling. Biochem. Biophys. Res. Commun..

[67] Hansen S., Hupp T.R., Lane D.P. (1996). Allosteric regulation of the thermostability and DNA binding activity of human p53 by specific interacting proteins. CRC Cell Transformation Group. J. Biol. Chem..

[68] Hupp T.R., Meek D.W., Midgley C.A., Lane D.P. (1992). Regulation of the specific DNA binding function of p53. Cell.

[69] Walerych D., Olszewski M.B., Gutkowska M., Helwak A., Zylicz M., Zylicz A. (2009). Hsp70 molecular chaperones are required to support p53 tumor suppressor activity under stress conditions. Oncogene.

[70] Wiech M., Olszewski M.B., Tracz-Gaszewska Z., Wawrzynow B., Zylicz M., Zylicz A. (2012). Molecular mechanism of mutant p53 stabilization: The role of HSP70 and MDM2. PloS ONE.

[71] Akakura S., Yoshida M., Yoneda Y., Horinouchi S. (2001). A role for Hsc70 in regulating nucleocytoplasmic transport of a temperature-sensitive p53 (p53Val-135). J. Biol. Chem..

[72] Rohde M., Daugaard M., Jensen M.H., Helin K., Nylandsted J., Jäättelä M. (2005). Members of the heat-shock protein 70 family promote cancer cell growth by distinct mechanisms. Genes Dev..

[73] Boysen M., Kityk R., Mayer M.P. (2019). Hsp70- and Hsp90-Mediated Regulation of the Conformation of p53 DNA Binding Domain and p53 Cancer Variants. Mol. Cell.

[74] Dahiya V., Agam G., Lawatscheck J., Rutz D.A., Lamb D.C., Buchner J. (2019). Coordinated Conformational Processing of the Tumor Suppressor Protein p53 by the Hsp70 and Hsp90 Chaperone Machineries. Mol. Cell.

[75] Wang N., Wang Z., Peng C., You J., Shen J., Han S., Chen J. (2014). Dietary compound isoliquiritigenin targets GRP78 to chemosensitize breast cancer stem cells via β-catenin/ABCG2 signaling. Carcinogenesis.

[76] Li Z., Wang Y., Wu H., Zhang L., Yang P., Li Z. (2014). GRP78 enhances the glutamine metabolism to support cell survival from glucose deficiency by modulating the β-catenin signaling. Oncotarget.

[77] Wei B., Cao J., Tian J.H., Yu C.Y., Huang Q., Yu J.J., Ma R., Wang J., Xu F., Wang L.B. (2021). Mortalin maintains breast cancer stem cells stemness via activation of Wnt/GSK3β/β-catenin signaling pathway. Am. J. Cancer Res..

[78] Zhang W., Xue D., Yin H., Wang S., Li C., Chen E., Hu D., Tao Y., Yu J., Zheng Q. (2016). Overexpression of HSPA1A enhances the osteogenic differentiation of bone marrow mesenchymal stem cells via activation of the Wnt/β-catenin signaling pathway. Sci. Rep..

[79] Ikezaki M., Higashimoto N., Matsumura K., Ihara Y. (2016). Hsc70 facilitates TGF-β-induced activation of Smad2/3 in fibroblastic NRK-49F cells. Biochem. Biophys. Res. Commun..

[80] Zhang L., Li Z., Fan Y., Li H., Li Z., Li Y. (2015). Overexpressed GRP78 affects EMT and cell-matrix adhesion via autocrine TGF-β/Smad2/3 signaling. Int. J. Biochem. Cell Biol..

[81] Li Y., Kang X., Wang Q. (2011). HSP70 decreases receptor-dependent phosphorylation of Smad2 and blocks TGF-β-induced epithelial-mesenchymal transition. J. Genet. Genom.=Yi Chuan Xue Bao.

[82] Liu J., Bao J., Hao J., Peng Y., Hong F. (2014). HSP70 inhibits high glucose-induced Smad3 activation and attenuates epithelial-to-mesenchymal transition of peritoneal mesothelial cells. Mol. Med. Rep..

[83] Zhou Y., Mao H., Li S., Cao S., Li Z., Zhuang S., Fan J., Dong X., Borkan S.C., Wang Y. (2010). HSP72 inhibits Smad3 activation and nuclear translocation in renal epithelial-to-mesenchymal transition. J. Am. Soc. Nephrol. JASN.

[84] Boudesco C., Cause S., Jego G., Garrido C. (2018). Hsp70: A Cancer Target Inside and Outside the Cell. Methods Mol. Biol. (Clifton N.J.).

[85] Sheppard P.W., Sun X., Khammash M., Giffard R.G. (2014). Overexpression of heat shock protein 72 attenuates NF-κB activation using a combination of regulatory mechanisms in microglia. PLoS Comput. Biol..

[86] Cho H.J., Gee H.Y., Baek K.H., Ko S.K., Park J.M., Lee H., Kim N.D., Lee M.G., Shin I. (2011). A small molecule that binds to an ATPase domain of Hsc70 promotes membrane trafficking of mutant cystic fibrosis transmembrane conductance regulator. J. Am. Chem. Soc..

[87] Macias A.T., Williamson D.S., Allen N., Borgognoni J., Clay A., Daniels Z., Dokurno P., Drysdale M.J., Francis G.L., Graham C.J. (2011). Adenosine-derived inhibitors of 78 kDa glucose regulated protein (Grp78) ATPase: Insights into isoform selectivity. J. Med. Chem..

[88] Guerrero-Juarez C.F., Lee G.H., Liu Y., Wang S., Karikomi M., Sha Y., Chow R.Y., Nguyen T.T.L., Iglesias V.S., Aasi S. (2022). Single-cell analysis of human basal cell carcinoma reveals novel regulators of tumor growth and the tumor microenvironment. Sci. Adv..

[89] Sakai K., Inoue M., Mikami S., Nishimura H., Kuwabara Y., Kojima A., Toda M., Ogawa-Kobayashi Y., Kikuchi S., Hirata Y. (2021). Functional inhibition of heat shock protein 70 by VER-155008 suppresses pleural mesothelioma cell proliferation via an autophagy mechanism. Thorac. Cancer.

[90] Brünnert D., Langer C., Zimmermann L., Bargou R.C., Burchardt M., Chatterjee M., Stope M.B. (2020). The heat shock protein 70 inhibitor VER155008 suppresses the expression of HSP27, HOP and HSP90β and the androgen receptor, induces apoptosis, and attenuates prostate cancer cell growth. J. Cell. Biochem..

[91] Dong J., Wu Z., Wang D., Pascal L.E., Nelson J.B., Wipf P., Wang Z. (2019). Hsp70 Binds to the Androgen Receptor N-terminal Domain and Modulates the Receptor Function in Prostate Cancer Cells. Mol. Cancer Ther..

[92] Kita K., Shiota M., Tanaka M., Otsuka A., Matsumoto M., Kato M., Tamada S., Iwao H., Miura K., Nakatani T. (2017). Heat shock protein 70 inhibitors suppress androgen receptor expression in LNCaP95 prostate cancer cells. Cancer Sci..

[93] Kim S.H., Kang J.G., Kim C.S., Ihm S.H., Choi M.G., Yoo H.J., Lee S.J. (2014). The hsp70 inhibitor VER155008 induces paraptosis requiring de novo protein synthesis in anaplastic thyroid carcinoma cells. Biochem. Biophys. Res. Commun..

[94] Massey A.J., Williamson D.S., Browne H., Murray J.B., Dokurno P., Shaw T., Macias A.T., Daniels Z., Geoffroy S., Dopson M. (2010). A novel, small molecule inhibitor of Hsc70/Hsp70 potentiates Hsp90 inhibitor induced apoptosis in HCT116 colon carcinoma cells. Cancer Chemother. Pharmacol..

[95] Ding L.X., Zhang J., Yang S.S., Wu J., Su T., Wang W.M. (2022). Heat Shock Proteins 70 Regulate Cell Motility and Invadopodia-Associated Proteins Expression in Oral Squamous Cell Carcinoma. Front. Endocrinol..

[96] Ko S.K., Kim J., Na D.C., Park S., Park S.H., Hyun J.Y., Baek K.H., Kim N.D., Kim N.K., Park Y.N. (2015). A small molecule inhibitor of ATPase activity of HSP70 induces apoptosis and has antitumor activities. Chem. Biol..

[97] Park S.H., Baek K.H., Shin I., Shin I. (2018). Subcellular Hsp70 Inhibitors Promote Cancer Cell Death via Different Mechanisms. Cell Chem. Biol..

[98] Tran P.L., Kim S.A., Choi H.S., Yoon J.H., Ahn S.G. (2010). Epigallocatechin-3-gallate suppresses the expression of HSP70 and HSP90 and exhibits anti-tumor activity in vitro and in vivo. BMC Cancer.

[99] Ermakova S.P., Kang B.S., Choi B.Y., Choi H.S., Schuster T.F., Ma W.Y., Bode A.M., Dong Z. (2006). (-)-Epigallocatechin gallate overcomes resistance to etoposide-induced cell death by targeting the molecular chaperone glucose-regulated protein 78. Cancer Res..

[100] Zeng Y., Cao R., Zhang T., Li S., Zhong W. (2015). Design and synthesis of piperidine derivatives as novel human heat shock protein 70 inhibitors for the treatment of drug-resistant tumors. Eur. J. Med. Chem..

[101] Fiengo L., Lauro G., Bellone M.L., Bifulco G., Dal Piaz F., De Tommasi N. (2021). The plant diterpene epoxysiderol targets Hsp70 in cancer cells, affecting its ATPase activity and reducing its translocation to plasma membrane. Int. J. Biol. Macromol..

[102] Rérole A.L., Gobbo J., De Thonel A., Schmitt E., Pais de Barros J.P., Hammann A., Lanneau D., Fourmaux E., Demidov O.N., Micheau O. (2011). Peptides and aptamers targeting HSP70: A novel approach for anticancer chemotherapy. Cancer Res..

[103] Rodina A., Patel P.D., Kang Y., Patel Y., Baaklini I., Wong M.J., Taldone T., Yan P., Yang C., Maharaj R. (2013). Identification of an allosteric pocket on human hsp70 reveals a mode of inhibition of this therapeutically important protein. Chem. Biol..

[104] Song T., Guo Y., Xue Z., Guo Z., Wang Z., Lin D., Zhang H., Pan H., Zhang X., Yin F. (2021). Small-molecule inhibitor targeting the Hsp70-Bim protein-protein interaction in CML cells overcomes BCR-ABL-independent TKI resistance. Leukemia.

[105] Zhang H., Song T., Wang Z., Laura Bonnette U., Guo Y., Wang H., Gao Q., Zhang Z. (2022). Bcr-Abl drives the formation of Hsp70/Bim PPI to stabilize oncogenic clients and prevent cells from undergoing apoptosis. Biochem. Pharmacol..

[106] Wang Z., Song T., Guo Z., Uwituze L.B., Guo Y., Zhang H., Wang H., Zhang X., Pan H., Ji T. (2021). A novel Hsp70 inhibitor specifically targeting the cancer-related Hsp70-Bim protein-protein interaction. Eur. J. Med. Chem..

[107] Petit T., Izbicka E., Lawrence R.A., Nalin C., Weitman S.D., Von Hoff D.D. (1999). Activity of MKT 077, a rhodacyanine dye, against human tumor colony-forming units. Anti-Cancer Drugs.

[108] Wadhwa R., Sugihara T., Yoshida A., Nomura H., Reddel R.R., Simpson R., Maruta H., Kaul S.C. (2000). Selective toxicity of MKT-077 to cancer cells is mediated by its binding to the hsp70 family protein mot-2 and reactivation of p53 function. Cancer Res..

[109] Rousaki A., Miyata Y., Jinwal U.K., Dickey C.A., Gestwicki J.E., Zuiderweg E.R. (2011). Allosteric drugs: The interaction of antitumor compound MKT-077 with human Hsp70 chaperones. J. Mol. Biol..

[110] Deocaris C.C., Widodo N., Shrestha B.G., Kaur K., Ohtaka M., Yamasaki K., Kaul S.C., Wadhwa R. (2007). Mortalin sensitizes human cancer cells to MKT-077-induced senescence. Cancer Lett..

[111] Colvin T.A., Gabai V.L., Gong J., Calderwood S.K., Li H., Gummuluru S., Matchuk O.N., Smirnova S.G., Orlova N.V., Zamulaeva I.A. (2014). Hsp70-Bag3 interactions regulate cancer-related signaling networks. Cancer Res..

[112] Wang A.M., Miyata Y., Klinedinst S., Peng H.M., Chua J.P., Komiyama T., Li X., Morishima Y., Merry D.E., Pratt W.B. (2013). Activation of Hsp70 reduces neurotoxicity by promoting polyglutamine protein degradation. Nat. Chem. Biol..

[113] Miyata Y., Li X., Lee H.F., Jinwal U.K., Srinivasan S.R., Seguin S.P., Young Z.T., Brodsky J.L., Dickey C.A., Sun D. (2013). Synthesis and initial evaluation of YM-08, a blood-brain barrier permeable derivative of the heat shock protein 70 (Hsp70) inhibitor MKT-077, which reduces tau levels. ACS Chem. Neurosci..

[114] Wu P.K., Hong S.K., Chen W., Becker A.E., Gundry R.L., Lin C.W., Shao H., Gestwicki J.E., Park J.I. (2020). Mortalin (HSPA9) facilitates BRAF-mutant tumor cell survival by suppressing ANT3-mediated mitochondrial membrane permeability. Sci. Signal..

[115] Li X., Srinivasan S.R., Connarn J., Ahmad A., Young Z.T., Kabza A.M., Zuiderweg E.R., Sun D., Gestwicki J.E. (2013). Analogs of the Allosteric Heat Shock Protein 70 (Hsp70) Inhibitor, MKT-077, as Anti-Cancer Agents. ACS Med. Chem. Lett..

[116] Li X., Colvin T., Rauch J.N., Acosta-Alvear D., Kampmann M., Dunyak B., Hann B., Aftab B.T., Murnane M., Cho M. (2015). Validation of the Hsp70-Bag3 protein-protein interaction as a potential therapeutic target in cancer. Mol. Cancer Ther..

[117] Shao H., Gestwicki J.E. (2020). Neutral analogs of the heat shock protein 70 (Hsp70) inhibitor, JG-98. Bioorganic Med. Chem. Lett..

[118] Shao H., Li X., Moses M.A., Gilbert L.A., Kalyanaraman C., Young Z.T., Chernova M., Journey S.N., Weissman J.S., Hann B. (2018). Exploration of Benzothiazole Rhodacyanines as Allosteric Inhibitors of Protein-Protein Interactions with Heat Shock Protein 70 (Hsp70). J. Med. Chem..

[119] Howe M.K., Bodoor K., Carlson D.A., Hughes P.F., Alwarawrah Y., Loiselle D.R., Jaeger A.M., Darr D.B., Jordan J.L., Hunter L.M. (2014). Identification of an allosteric small-molecule inhibitor selective for the inducible form of heat shock protein 70. Chem. Biol..

[120] Adam C., Baeurle A., Brodsky J.L., Wipf P., Schrama D., Becker J.C., Houben R. (2014). The HSP70 modulator MAL3-101 inhibits Merkel cell carcinoma. PloS ONE.

[121] Braunstein M.J., Scott S.S., Scott C.M., Behrman S., Walter P., Wipf P., Coplan J.D., Chrico W., Joseph D., Brodsky J.L. (2011). Antimyeloma Effects of the Heat Shock Protein 70 Molecular Chaperone Inhibitor MAL3-101. J. Oncol..

[122] Fewell S.W., Smith C.M., Lyon M.A., Dumitrescu T.P., Wipf P., Day B.W., Brodsky J.L. (2004). Small molecule modulators of endogenous and co-chaperone-stimulated Hsp70 ATPase activity. J. Biol. Chem..

[123] Huryn D.M., Brodsky J.L., Brummond K.M., Chambers P.G., Eyer B., Ireland A.W., Kawasumi M., Laporte M.G., Lloyd K., Manteau B. (2011). Chemical methodology as a source of small-molecule checkpoint inhibitors and heat shock protein 70 (Hsp70) modulators. Proc. Natl. Acad. Sci. USA.

[124] Zhou Y., Ma J., Zhang J., He L., Gong J., Long C. (2017). Pifithrin-μ is efficacious against non-small cell lung cancer via inhibition of heat shock protein 70. Oncol. Rep..

[125] Leu J.I., Pimkina J., Pandey P., Murphy M.E., George D.L. (2011). HSP70 inhibition by the small-molecule 2-phenylethynesulfonamide impairs protein clearance pathways in tumor cells. Molecular Cancer Res. MCR.

[126] Kaiser M., Kühnl A., Reins J., Fischer S., Ortiz-Tanchez J., Schlee C., Mochmann L.H., Heesch S., Benlasfer O., Hofmann W.K. (2011). Antileukemic activity of the HSP70 inhibitor pifithrin-μ in acute leukemia. Blood Cancer J..

[127] Leu J.I., Pimkina J., Frank A., Murphy M.E., George D.L. (2009). A small molecule inhibitor of inducible heat shock protein 70. Mol. Cell.

[128] Leu J.I., Zhang P., Murphy M.E., Marmorstein R., George D.L. (2014). Structural basis for the inhibition of HSP70 and DnaK chaperones by small-molecule targeting of a C-terminal allosteric pocket. ACS Chem. Biol..

[129] Balaburski G.M., Leu J.I., Beeharry N., Hayik S., Andrake M.D., Zhang G., Herlyn M., Villanueva J., Dunbrack R.L., Yen T. (2013). A modified HSP70 inhibitor shows broad activity as an anticancer agent. Mol. Cancer Res. MCR.

[130] Bailey C.K., Budina-Kolomets A., Murphy M.E., Nefedova Y. (2015). Efficacy of the HSP70 inhibitor PET-16 in multiple myeloma. Cancer Biol. Ther..

[131] Barnoud T., Leung J.C., Leu J.I., Basu S., Poli A.N.R., Parris J.L.D., Indeglia A., Martynyuk T., Good M., Gnanapradeepan K. (2020). A Novel Inhibitor of HSP70 Induces Mitochondrial Toxicity and Immune Cell Recruitment in Tumors. Cancer Res..

[132] Hassan A.Q., Kirby C.A., Zhou W., Schuhmann T., Kityk R., Kipp D.R., Baird J., Chen J., Chen Y., Chung F. (2015). The novolactone natural product disrupts the allosteric regulation of Hsp70. Chem. Biol..

[133] Ambrose A.J., Zerio C.J., Sivinski J., Schmidlin C.J., Shi T., Ross A.B., Widrick K.J., Johnson S.M., Zhang D.D., Chapman E. (2019). A high throughput substrate binding assay reveals hexachlorophene as an inhibitor of the ER-resident HSP70 chaperone GRP78. Bioorganic Med. Chem. Lett..

[134] Schmitt E., Maingret L., Puig P.E., Rerole A.L., Ghiringhelli F., Hammann A., Solary E., Kroemer G., Garrido C. (2006). Heat shock protein 70 neutralization exerts potent antitumor effects in animal models of colon cancer and melanoma. Cancer Res..

[135] Schmitt E., Parcellier A., Gurbuxani S., Cande C., Hammann A., Morales M.C., Hunt C.R., Dix D.J., Kroemer R.T., Giordanetto F. (2003). Chemosensitization by a non-apoptogenic heat shock protein 70-binding apoptosis-inducing factor mutant. Cancer Res..

[136] Ernst K., Liebscher M., Mathea S., Granzhan A., Schmid J., Popoff M.R., Ihmels H., Barth H., Schiene-Fischer C. (2016). A novel Hsp70 inhibitor prevents cell intoxication with the actin ADP-ribosylating Clostridium perfringens iota toxin. Sci. Rep..

[137] Nadler S.G., Dischino D.D., Malacko A.R., Cleaveland J.S., Fujihara S.M., Marquardt H. (1998). Identification of a binding site on Hsc70 for the immunosuppressant 15-deoxyspergualin. Biochem. Biophys. Res. Commun..

[138] Kawada M., Someno T., Inuma H., Masuda T., Ishizuka M., Takeuchi T. (2000). The long-lasting antiproliferative effect of 15-deoxyspergualin through its spermidine moiety. J. Antibiot..

[139] Kawada M., Masuda T., Ishizuka M., Takeuchi T. (2002). 15-Deoxyspergualin inhibits Akt kinase activation and phosphatidylcholine synthesis. J. Biol. Chem..

[140] Ramya T.N., Surolia N., Surolia A. (2006). 15-Deoxyspergualin modulates Plasmodium falciparum heat shock protein function. Biochem. Biophys. Res. Commun..

[141] Cerezo M., Lehraiki A., Millet A., Rouaud F., Plaisant M., Jaune E., Botton T., Ronco C., Abbe P., Amdouni H. (2016). Compounds Triggering ER Stress Exert Anti-Melanoma Effects and Overcome BRAF Inhibitor Resistance. Cancer Cell.

[142] Ambrose A.J., Santos E.A., Jimenez P.C., Rocha D.D., Wilke D.V., Beuzer P., Axelrod J., Kumar Kanduluru A., Fuchs P.L., Cang H. (2017). Ritterostatin G(N) 1(N), a Cephalostatin-Ritterazine Bis-steroidal Pyrazine Hybrid, Selectively Targets GRP78. Chembiochem A Eur. J. Chem. Biol..

[143] Mikeladze M.A., Dutysheva E.A., Kartsev V.G., Margulis B.A., Guzhova I.V., Lazarev V.F. (2021). Disruption of the Complex between GAPDH and Hsp70 Sensitizes C6 Glioblastoma Cells to Hypoxic Stress. Int. J. Mol. Sci..

[144] Sverchinsky D.V., Nikotina A.D., Komarova E.Y., Mikhaylova E.R., Aksenov N.D., Lazarev V.F., Mitkevich V.A., Suezov R., Druzhilovskiy D.S., Poroikov V.V. (2018). Etoposide-Induced Apoptosis in Cancer Cells Can Be Reinforced by an Uncoupled Link between Hsp70 and Caspase-3. Int. J. Mol. Sci..

[145] Phillips P.A., Dudeja V., McCarroll J.A., Borja-Cacho D., Dawra R.K., Grizzle W.E., Vickers S.M., Saluja A.K. (2007). Triptolide induces pancreatic cancer cell death via inhibition of heat shock protein 70. Cancer Res..

[146] Oliveira A., Beyer G., Chugh R., Skube S.J., Majumder K., Banerjee S., Sangwan V., Li L., Dawra R., Subramanian S. (2015). Triptolide abrogates growth of colon cancer and induces cell cycle arrest by inhibiting transcriptional activation of E2F. Lab. Investig. A J. Tech. Methods Pathol..

[147] Banerjee S., Thayanithy V., Sangwan V., Mackenzie T.N., Saluja A.K., Subramanian S. (2013). Minnelide reduces tumor burden in preclinical models of osteosarcoma. Cancer Lett..

[148] Antonoff M.B., Chugh R., Skube S.J., Dudeja V., Borja-Cacho D., Clawson K.A., Vickers S.M., Saluja A.K. (2010). Role of Hsp-70 in triptolide-mediated cell death of neuroblastoma. J. Surg. Res..

[149] MacKenzie T.N., Mujumdar N., Banerjee S., Sangwan V., Sarver A., Vickers S., Subramanian S., Saluja A.K. (2013). Triptolide induces the expression of miR-142-3p: A negative regulator of heat shock protein 70 and pancreatic cancer cell proliferation. Mol. Cancer Ther..

[150] Jacobson B.A., Chen E.Z., Tang S., Belgum H.S., McCauley J.A., Evenson K.A., Etchison R.G., Jay-Dixon J., Patel M.R., Raza A. (2015). Triptolide and its prodrug minnelide suppress Hsp70 and inhibit in vivo growth in a xenograft model of mesothelioma. Genes Cancer.

[151] Rousalova I., Banerjee S., Sangwan V., Evenson K., McCauley J.A., Kratzke R., Vickers S.M., Saluja A., D’Cunha J. (2013). Minnelide: A novel therapeutic that promotes apoptosis in non-small cell lung carcinoma in vivo. PloS ONE.

[152] Chugh R., Sangwan V., Patil S.P., Dudeja V., Dawra R.K., Banerjee S., Schumacher R.J., Blazar B.R., Georg G.I., Vickers S.M. (2012). A preclinical evaluation of Minnelide as a therapeutic agent against pancreatic cancer. Sci. Transl. Med..

[153] Tsai C.H., Weng J.R., Lin H.W., Lu M.T., Liu Y.C., Chu P.C. (2022). Targeting Triple Negative Breast Cancer Stem Cells by Heat Shock Protein 70 Inhibitors. Cancers.

[154] Chang C.S., Kumar V., Lee D.Y., Chen Y., Wu Y.C., Gao J.Y., Chu P.C. (2021). Development of Novel Rhodacyanine-Based Heat Shock Protein 70 Inhibitors. Curr. Med. Chem..

[155] Guo Z., Song T., Wang Z., Lin D., Cao K., Liu P., Feng Y., Zhang X., Wang P., Yin F. (2020). The chaperone Hsp70 is a BH3 receptor activated by the pro-apoptotic Bim to stabilize anti-apoptotic clients. J. Biol. Chem..

[156] Sannino S., Yates M.E., Schurdak M.E., Oesterreich S., Lee A.V., Wipf P., Brodsky J.L. (2021). Unique integrated stress response sensors regulate cancer cell susceptibility when Hsp70 activity is compromised. eLife.

[157] Sannino S., Guerriero C.J., Sabnis A.J., Stolz D.B., Wallace C.T., Wipf P., Watkins S.C., Bivona T.G., Brodsky J.L. (2018). Compensatory increases of select proteostasis networks after Hsp70 inhibition in cancer cells. J. Cell Sci..

[158] Freeman B.C., Myers M.P., Schumacher R., Morimoto R.I. (1995). Identification of a regulatory motif in Hsp70 that affects ATPase activity, substrate binding and interaction with HDJ-1. EMBO J..

[159] Lazarev V.F., Sverchinsky D.V., Mikhaylova E.R., Semenyuk P.I., Komarova E.Y., Niskanen S.A., Nikotina A.D., Burakov A.V., Kartsev V.G., Guzhova I.V. (2018). Sensitizing tumor cells to conventional drugs: HSP70 chaperone inhibitors, their selection and application in cancer models. Cell Death Dis..

[160] Zhang H., Guo Z., Guo Y., Wang Z., Tang Y., Song T., Zhang Z. (2021). Bim transfer between Bcl-2-like protein and Hsp70 underlines Bcl-2/Hsp70 crosstalk to regulate apoptosis. Biochem. Pharmacol..

[161] Moses M.A., Kim Y.S., Rivera-Marquez G.M., Oshima N., Watson M.J., Beebe K.E., Wells C., Lee S., Zuehlke A.D., Shao H. (2018). Targeting the Hsp40/Hsp70 Chaperone Axis as a Novel Strategy to Treat Castration-Resistant Prostate Cancer. Cancer Res..

[162] Park S.H., Kim W.J., Li H., Seo W., Park S.H., Kim H., Shin S.C., Zuiderweg E.R.P., Kim E.E., Sim T. (2017). Anti-leukemia activity of a Hsp70 inhibitor and its hybrid molecules. Sci. Rep..

[163] Cavanaugh A., Juengst B., Sheridan K., Danella J.F., Williams H. (2015). Combined inhibition of heat shock proteins 90 and 70 leads to simultaneous degradation of the oncogenic signaling proteins involved in muscle invasive bladder cancer. Oncotarget.

[164] Miyagawa T., Saito H., Minamiya Y., Mitobe K., Takashima S., Takahashi N., Ito A., Imai K., Motoyama S., Ogawa J. (2014). Inhibition of Hsp90 and 70 sensitizes melanoma cells to hyperthermia using ferromagnetic particles with a low Curie temperature. Int. J. Clin. Oncol..

[165] Ma L., Sato F., Sato R., Matsubara T., Hirai K., Yamasaki M., Shin T., Shimada T., Nomura T., Mori K. (2014). Dual targeting of heat shock proteins 90 and 70 promotes cell death and enhances the anticancer effect of chemotherapeutic agents in bladder cancer. Oncol. Rep..

[166] Kim S.H., Kang J.G., Kim C.S., Ihm S.H., Choi M.G., Yoo H.J., Lee S.J. (2014). Hsp70 inhibition potentiates radicicol-induced cell death in anaplastic thyroid carcinoma cells. AntiCancer Res..

[167] Reikvam H., Nepstad I., Sulen A., Gjertsen B.T., Hatfield K.J., Bruserud Ø. (2013). Increased antileukemic effects in human acute myeloid leukemia by combining HSP70 and HSP90 inhibitors. Expert Opin. Investig. Drugs.

[168] McKeon A.M., Egan A., Chandanshive J., McMahon H., Griffith D.M. (2016). Novel Improved Synthesis of HSP70 Inhibitor, Pifithrin-μ. In Vitro Synergy Quantification of Pifithrin-μ Combined with Pt Drugs in Prostate and Colorectal Cancer Cells. Molecules.

[169] Liu J., Liu J., Guo S.Y., Liu H.L., Li S.Z. (2017). HSP70 inhibitor combined with cisplatin suppresses the cervical cancer proliferation in vitro and transplanted tumor growth: An experimental study. Asian Pac. J. Trop. Med..

[170] Liu J., Liu J., Li S.Z., Zheng Y.A., Guo S.Y., Wang X. (2016). [Inhibiting HSP70 expression enhances cisplatin sensitivity of cervical cancer cells. Nan Fang Yi Ke Da Xue Xue Bao=J. South. Med. Univ..

[171] Alsaied O.A., Sangwan V., Banerjee S., Krosch T.C., Chugh R., Saluja A., Vickers S.M., Jensen E.H. (2014). Sorafenib and triptolide as combination therapy for hepatocellular carcinoma. Surgery.

[172] Budina-Kolomets A., Webster M.R., Leu J.I., Jennis M., Krepler C., Guerrini A., Kossenkov A.V., Xu W., Karakousis G., Schuchter L. (2016). HSP70 Inhibition Limits FAK-Dependent Invasion and Enhances the Response to Melanoma Treatment with BRAF Inhibitors. Cancer Res..

[173] Huang L., Wang Y., Bai J., Yang Y., Wang F., Feng Y., Zhang R., Li F., Zhang P., Lv N. (2020). Blockade of HSP70 by VER-155008 synergistically enhances bortezomib-induced cytotoxicity in multiple myeloma. Cell Stress Chaperones.

[174] Yerlikaya A., Okur E., Eker S., Erin N. (2010). Combined effects of the proteasome inhibitor bortezomib and Hsp70 inhibitors on the B16F10 melanoma cell line. Mol. Med. Rep..

[175] Xu J., Wang K., Zhang X., Qiu Y., Huang D., Li W., Xiao X., Tian Y. (2010). HSP70: A promising target for laryngeal carcinoma radiaotherapy by inhibiting cleavage and degradation of nucleolin. J. Exp. Clin. Cancer Res. CR.

[176] Kumar S., Stokes J., Singh U.P., Scissum Gunn K., Acharya A., Manne U., Mishra M. (2016). Targeting Hsp70: A possible therapy for cancer. Cancer Lett..

[177] Li C., Zhang B., Lv W., Lai C., Chen Z., Wang R., Long X., Feng X. (2016). Triptolide inhibits cell growth and GRP78 protein expression but induces cell apoptosis in original and radioresistant NPC cells. Oncotarget.

[178] Xia Y., Li C., Cao J., Chen Z., Wang J., Wu Y., Zhang X. (2022). Liposome-templated gold nanoparticles for precisely temperature-controlled photothermal therapy based on heat shock protein expression. Colloids Surfaces. B Biointerfaces.

[179] Liu C., Qin H., Kang L., Chen Z., Wang H., Qiu H., Ren J., Qu X. (2018). Graphitic carbon nitride nanosheets as a multifunctional nanoplatform for photochemical internalization-enhanced photodynamic therapy. J. Mater. Chem. B.

[180] Yang W., Cui M., Lee J., Gong W., Wang S., Fu J., Wu G., Yan K. (2016). Heat shock protein inhibitor, quercetin, as a novel adjuvant agent to improve radiofrequency ablation-induced tumor destruction and its molecular mechanism. Chin. J. Cancer Res.=Chung-Kuo Yen Cheng Yen Chiu.

[181] Zhong Y., Zou Y., Liu L., Li R., Xue F., Yi T. (2020). pH-responsive Ag(2)S nanodots loaded with heat shock protein 70 inhibitor for photoacoustic imaging-guided photothermal cancer therapy. Acta Biomater..

[182] Song H.B. (2019). Possible involvement of HSP70 in pancreatic cancer cell proliferation after heat exposure and impact on RFA postoperative patient prognosis. Biochem. Biophys. Rep..

[183] Yang L., Tseng Y.T., Suo G., Chen L., Yu J., Chiu W.J., Huang C.C., Lin C.H. (2015). Photothermal therapeutic response of cancer cells to aptamer-gold nanoparticle-hybridized graphene oxide under NIR illumination. ACS Appl. Mater. Interfaces.

[184] Garrod T., Grubor-Bauk B., Yu S., Gargett T., Gowans E.J. (2014). Encoded novel forms of HSP70 or a cytolytic protein increase DNA vaccine potency. Hum. Vaccines Immunother..

[185] Li J., Xing Y., Zhou Z., Yao W., Cao R., Li T., Xu M., Wu J. (2016). Microbial HSP70 peptide epitope 407-426 as adjuvant in tumor-derived autophagosome vaccine therapy of mouse lung cancer. Tumour Biol.

[186] Han L., Wang W., Lu J., Kong F., Ma G., Zhu Y., Zhao D., Zhu J., Shuai W., Zhou Q. (2014). AAV-sBTLA facilitates HSP70 vaccine-triggered prophylactic antitumor immunity against a murine melanoma pulmonary metastasis model in vivo. Cancer Lett..

[187] Han L., Wang W., Fang Y., Feng Z., Liao S., Li W., Li Y., Li C., Maitituoheti M., Dong H. (2009). Soluble B and T lymphocyte attenuator possesses antitumor effects and facilitates heat shock protein 70 vaccine-triggered antitumor immunity against a murine TC-1 cervical cancer model in vivo. J. Immunol..

[188] Wu Y., Wan T., Zhou X., Wang B., Yang F., Li N., Chen G., Dai S., Liu S., Zhang M. (2005). Hsp70-like protein 1 fusion protein enhances induction of carcinoembryonic antigen-specific CD8+ CTL response by dendritic cell vaccine. Cancer Res..

[189] Ge C., Xing Y., Wang Q., Xiao W., Lu Y., Hu X., Gao Z., Xu M., Ma Y., Cao R. (2011). Improved efficacy of therapeutic vaccination with dendritic cells pulsed with tumor cell lysate against hepatocellular carcinoma by introduction of 2 tandem repeats of microbial HSP70 peptide epitope 407-426 and OK-432. Int. Immunopharmacol..

[190] Soleimanjahi H., Razavinikoo H., Fotouhi F., Ardebili A. (2017). Antitumor Response to a Codon-Optimized HPV-16 E7/HSP70 Fusion Antigen DNA Vaccine. Iran. J. Immunol. IJI.

[191] Zong J., Wang C., Wang Q., Peng Q., Xu Y., Xie X., Xu X. (2013). HSP70 and modified HPV 16 E7 fusion gene without the addition of a signal peptide gene sequence as a candidate therapeutic tumor vaccine. Oncol. Rep..

[192] Zong J., Wang C., Liu B., Liu M., Cao Y., Sun X., Yao Y., Sun G. (2013). Human hsp70 and HPV16 oE7 fusion protein vaccine induces an effective antitumor efficacy. Oncol. Rep..

[193] Farzanehpour M., Soleimanjahi H., Hassan Z.M., Amanzadeh A., Ghaemi A., Fazeli M. (2013). HSP70 modified response against HPV based tumor. Eur. Rev. Med. Pharmacol. Sci..

[194] Zong J., Peng Q., Wang Q., Zhang T., Fan D., Xu X. (2009). Human HSP70 and modified HPV16 E7 fusion DNA vaccine induces enhanced specific CD8+ T cell responses and anti-tumor effects. Oncol. Rep..

[195] Li H., Ou X., Xiong J. (2007). Modified HPV16 E7/HSP70 DNA vaccine with high safety and enhanced cellular immunity represses murine lung metastatic tumors with downregulated expression of MHC class I molecules. Gynecol. Oncol..

[196] Hauser H., Chen S.Y. (2003). Augmentation of DNA vaccine potency through secretory heat shock protein-mediated antigen targeting. Methods.

[197] Chen C.H., Wang T.L., Hung C.F., Yang Y., Young R.A., Pardoll D.M., Wu T.C. (2000). Enhancement of DNA vaccine potency by linkage of antigen gene to an HSP70 gene. Cancer Res..

[198] Zhang X., Huang Y., Li X., Wang Y., Yuan Y., Li M. (2020). Preparation of a new combination nanoemulsion-encapsulated MAGE1-MAGE3-MAGEn/HSP70 vaccine and study of its immunotherapeutic effect. Pathol. Res. Pract..

[199] Long P., Zhang Q., Xue M., Cao G., Li C., Chen W., Jin F., Li Z., Li R., Wang X. (2019). Tomato lectin-modified nanoemulsion-encapsulated MAGE1-HSP70/SEA complex protein vaccine: Targeting intestinal M cells following peroral administration. Biomed. Pharmacother..

[200] Jiang J., Xie D., Zhang W., Xiao G., Wen J. (2013). Fusion of Hsp70 to Mage-a1 enhances the potency of vaccine-specific immune responses. J. Transl. Med..

[201] Ge W., Li Y., Li Z.S., Zhang S.H., Sun Y.J., Hu P.Z., Wang X.M., Huang Y., Si S.Y., Zhang X.M. (2009). The antitumor immune responses induced by nanoemulsion-encapsulated MAGE1-HSP70/SEA complex protein vaccine following peroral administration route. Cancer Immunol. Immunother. CII.

[202] Liu T.T., Wu Y., Niu T. (2018). Human DKK1 and human HSP70 fusion DNA vaccine induces an effective anti-tumor efficacy in murine multiple myeloma. Oncotarget.

[203] Lan Y.H., Li Y.G., Liang Z.W., Chen M., Peng M.L., Tang L., Hu H.D., Ren H. (2007). A DNA vaccine against chimeric AFP enhanced by HSP70 suppresses growth of hepatocellular carcinoma. Cancer Immunol. Immunother. CII.

[204] Lan Y.H., Li Y.G., Chen M., Tang L., Ren H. (2006). [Immunotherapy with a chimeric AFP and HSP70 gene DNA vaccine targeting on a murine hepatocellular carcinoma. Zhonghua Gan Zang Bing Za Zhi=Zhonghua Ganzangbing Zazhi=Chin. J. Hepatol..

[205] Dong L., Zhang X., Ren J., Wu S., Yu T., Hou L., Fu L., Yi S., Yu C. (2013). Human prostate stem cell antigen and HSP70 fusion protein vaccine inhibits prostate stem cell antigen-expressing tumor growth in mice. Cancer Biother. Radiopharm..

[206] Zhang X., Yu C., Zhao J., Fu L., Yi S., Liu S., Yu T., Chen W. (2007). Vaccination with a DNA vaccine based on human PSCA and HSP70 adjuvant enhances the antigen-specific CD8+ T-cell response and inhibits the PSCA+ tumors growth in mice. J. Gene Med..

[207] Zhang H., Huang W. (2006). Fusion proteins of Hsp70 with tumor-associated antigen acting as a potent tumor vaccine and the C-terminal peptide-binding domain of Hsp70 being essential in inducing antigen-independent anti-tumor response in vivo. Cell Stress Chaperones.

[208] Yamaoka A., Guan X., Takemoto S., Nishikawa M., Takakura Y. (2010). Development of a novel Hsp70-based DNA vaccine as a multifunctional antigen delivery system. J. Control. Release.

[209] Karyampudi L., Ghosh S.K. (2008). Mycobacterial HSP70 as an adjuvant in the design of an idiotype vaccine against a murine lymphoma. Cell. Immunol..

[210] Kim J.H., Majumder N., Lin H., Watkins S., Falo L.D., You Z. (2005). Induction of therapeutic antitumor immunity by in vivo administration of a lentiviral vaccine. Hum. Gene Ther..

[211] Xu M., Zhou L., Zhang Y., Xie Z., Zhang J., Guo L., Wang C., Yang X. (2015). A Fixed Human Umbilical Vein Endothelial Cell Vaccine With 2 Tandem Repeats of Microbial HSP70 Peptide Epitope 407-426 As Adjuvant for Therapy of Hepatoma in Mice. J. Immunother..

[212] Wang Z.Y., Xing Y., Liu B., Lu L., Huang X., Ge C.Y., Yao W.J., Xu M.L., Gao Z.Q., Cao R.Y. (2012). Protective antitumor immunity induced by tumor cell lysates conjugated with diphtheria toxin and adjuvant epitope in mouse breast tumor models. Chin. J. Cancer.

[213] Chen C.H., Wang T.L., Hung C.F., Pardoll D.M., Wu T.C. (2000). Boosting with recombinant vaccinia increases HPV-16 E7-specific T cell precursor frequencies of HPV-16 E7-expressing DNA vaccines. Vaccine.

[214] Brinkman J.A., Xu X., Kast W.M. (2007). The efficacy of a DNA vaccine containing inserted and replicated regions of the E7 gene for treatment of HPV-16 induced tumors. Vaccine.

[215] Propper D.J., Braybrooke J.P., Taylor D.J., Lodi R., Styles P., Cramer J.A., Collins W.C., Levitt N.C., Talbot D.C., Ganesan T.S. (1999). Phase I trial of the selective mitochondrial toxin MKT077 in chemo-resistant solid tumours. Ann. Oncol. Off. J. Eur. Soc. Med. Oncol..

[216] Matsui H.M., Hazama S., Nakajima M., Xu M., Matsukuma S., Tokumitsu Y., Shindo Y., Tomochika S., Yoshida S., Iida M. (2021). Novel adjuvant dendritic cell therapy with transfection of heat-shock protein 70 messenger RNA for patients with hepatocellular carcinoma: A phase I/II prospective randomized controlled clinical trial. Cancer Immunol. Immunother. CII.

[217] Nakajima M., Hazama S., Tamada K., Udaka K., Kouki Y., Uematsu T., Arima H., Saito A., Doi S., Matsui H. (2020). A phase I study of multi-HLA-binding peptides derived from heat shock protein 70/glypican-3 and a novel combination adjuvant of hLAG-3Ig and Poly-ICLC for patients with metastatic gastrointestinal cancers: YNP01 trial. Cancer Immunol. Immunother. CII.

[218] Krause S.W., Gastpar R., Andreesen R., Gross C., Ullrich H., Thonigs G., Pfister K., Multhoff G. (2004). Treatment of colon and lung cancer patients with ex vivo heat shock protein 70-peptide-activated, autologous natural killer cells: A clinical phase i trial. Clin. Cancer Res..

[219] Li Z., Qiao Y., Liu B., Laska E.J., Chakravarthi P., Kulko J.M., Bona R.D., Fang M., Hegde U., Moyo V. (2005). Combination of imatinib mesylate with autologous leukocyte-derived heat shock protein and chronic myelogenous leukemia. Clin. Cancer Res. Off. J. Am. Assoc. Cancer Res..

[220] Skorupan N., Ahmad M.I., Steinberg S.M., Trepel J.B., Cridebring D., Han H., Von Hoff D.D., Alewine C. (2022). A phase II trial of the super-enhancer inhibitor Minnelide™ in advanced refractory adenosquamous carcinoma of the pancreas. Future Oncol..

